# In Vitro Antioxidant Activity and In Vivo Neuroprotective Effect of *Parastrephia quadrangularis* in a *Drosophila* Parkinson’s Disease Model

**DOI:** 10.3390/antiox14101226

**Published:** 2025-10-12

**Authors:** Branco Cárdenas, Ayza Cuevas, Duxan Arancibia, Lucas Urrutia, Pedro Zamorano, Adrián Paredes, Rafaella V. Zárate

**Affiliations:** 1 Instituto Antofagasta (IA), Universidad de Antofagasta, Antofagasta 1271155, Chile; branco.cardenas.zeballos@ua.cl (B.C.); ayza.cuevas.salinas@ua.cl (A.C.); pedro.zamorano@uamail.cl (P.Z.); 2 Departamento de Ciencias Farmacéuticas, Facultad de Ciencias, Universidad Católica del Norte, Antofagasta 1270709, Chile; duxan.arancibia@ucn.cl; 3 Facultad de Medicina, Universidad San Sebastián, Santiago 7510157, Chile; lucas88413@gmail.com; 4 Facultad de Ciencias de la Salud, Universidad de Antofagasta, Antofagasta 1240000, Chile

**Keywords:** *Parastrephia quadrangularis*, flavonoids, antioxidant activity, Parkinson’s disease, *Drosophila melanogaster*

## Abstract

Oxidative stress (OxS) is a central factor in neurodegenerative diseases (NDs), including Parkinson’s disease (PD). Phenolic compounds, including flavonoids and coumarins, counteract reactive species and modulate key intracellular survival pathways, highlighting their therapeutic potential. *Parastrephia quadrangularis* (*Pq*), a plant from the Atacama Desert traditionally used by Andean communities, contains phenolic compounds with antioxidant, antifungal, and anti-inflammatory activities. However, its neuroprotective potential remains unexplored. Here, a hydroalcoholic extract (HAE) of *Pq* and four subfractions (MeOH, EtOAc, DCM, and n-hex) were obtained and assessed for in vitro antioxidant activity, with HAE selected for its consistent activity. In SH-SY5Y cells, HAE-*Pq* lowered basal reactive oxygen species and attenuated hydrogen peroxide-induced OxS. The UHPLC-MS analysis of HAE-*Pq* unveiled a high abundance of flavonoids, followed by coumarins and phenolic acids, and identified 16 additional metabolites, including jaceidin as the most abundant. In vivo assays using a *Drosophila* genetic PD model induced by overexpression of human α-synuclein, showed that HAE-*Pq* was non-toxic and non-aversive and that it delayed the onset of motor defects by one week in female flies. This study provides the first evidence of the neuroprotective potential of *Pq*, supporting its value as a source of bioactive metabolites relevant to NDs and reinforcing its ethnopharmacological validation.

## 1. Introduction

Cellular metabolism continuously produces reactive oxygen species (ROS) and reactive nitrogen species (RNS) as byproducts, which are essential for redox homeostasis and cell signaling processes such as immune response, blood pressure regulation, cognitive function, and gene expression [1,2,3,4]. An imbalance in their levels can lead to cellular damage and disease. The mitochondrial electron transport chain and NADPH oxidase are the main intracellular sources of ROS [5,6,7], while additional enzymatic systems (e.g., peroxidases, nitric oxide synthase, lipoxygenases, xanthine oxidases, and cytochrome P450 [8,9,10]) and exogenous factors (air pollution, radiation, xenobiotics, and certain drugs [11]) also contribute. Endogenous enzymatic and non-enzymatic antioxidant systems maintain their levels strictly controlled [12]. The disruption of this balance in either direction may produce detrimental cellular outcomes. Chronic accumulation of ROS and RNS results in oxidative and nitrosative stress, increasing the vulnerability for developing pathological processes, including neurodegenerative diseases (NDs).

Parkinson’s disease (PD) is a ND in which oxidative stress contributes to pathogenesis [13]. It is characterized by the progressive loss of dopaminergic neurons from the nigrostriatal pathway and the presence of toxic intracellular aggregates of α-synuclein (α-syn), known as Lewy bodies (LB). Motor symptoms appear once dopaminergic terminals are significantly lost and striatal dopamine (DA) decreases by 60–80% [14]. Before this, non-motor symptoms may precede the motor manifestation [15]. The aggregation of α-syn, mitochondrial dysfunction, disruption of proteasomal and lysosomal-autophagy degradation systems, ferroptosis, and neuroinflammation are other mechanisms involved in the pathogenesis, which may interact bidirectionally with oxidative stress driving neuronal death [16]. Current gold-standard treatments include DA replacement, which mitigates the motor symptoms but does not halt neurodegeneration. Since the early 2000s, phytochemicals have been explored as promising candidates for neuroprotection, capable of modulating multiple pathogenic mechanisms, including oxidative stress, mitochondrial dysfunction, accumulation of insoluble aggregates, and neuroinflammation, with the potential to function as a disease-modifying therapy [17].

Phytochemicals, such as polyphenols, exhibit diverse biological activities, including antioxidant, antimicrobial, anti-inflammatory, and antiproliferative effects [18]. Flavonoids, a large group of polyphenols, act as multifunctional phytochemicals directly scavenging reactive species [19] and as modulators of key intracellular pathways to reduce neuroinflammation, apoptosis and ferroptosis, protein aggregation, and oxidative stress [20]. Well-studied flavonoids (e.g., quercetin, kaempferol, apigenin, luteolin, and catechins) enhance PI3K/Akt/GSK-3β and Nrf2/ARE pathways, inhibit JNK/p38 and NF-kB cascades [21,22,23,24,25,26,27,28,29,30,31,32,33,34,35], and stimulate BDNF/Trk signaling [36,37,38,39,40]. In PD models, they protect dopaminergic neurons by increasing tyrosine hydroxylase (TH) activity and inhibiting monoamine oxidase (MAO). Also, the inhibition of MAO increases DA levels [41,42,43,44,45]. This evidence emphasizes the prospective use of flavonoids and other phytochemicals as therapeutic agents and supports ongoing efforts to search for new sources and structures of bioactive compounds.

**Figure 1:**
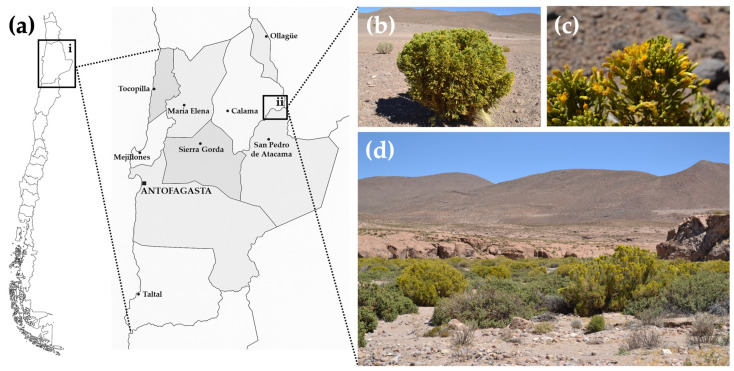
Geographic distribution and natural habitat of *Parastrephia quadrangularis*. (**a**) Map of Chile showing the location of the Antofagasta Region (inset i, black box) and the specific collection site near Quebrada de Chita (inset ii, black box), Atacama Desert. The specimen of *P. quadrangularis* was collected, in April 2022. (**b**) Image of aerial parts of *P. quadrangularis*, (**c**) close-up view of leaves and flowers, (**d**) shrub community with predominant presence of “Tola”, called “Tolar”. (Photographs obtained by Dr. Adrián Paredes).

*Parastrephia quadrangularis* (Meyen) Cabrera is a resinous shrub endemic to the Atacama Desert (Figure 1), located in the high Andean region at elevations above 3500 m above sea level (m.a.s.l.). Traditionally, high Andean communities have used it to treat digestive disorders, skeletal and muscular conditions, dermatological issues, respiratory and urinary infections, altitude sickness, and inflammatory diseases [46]. Diverse biological activities have been reported, encompassing hypotensive effects, gastroprotective activity, antifungal properties, and antioxidant activity [47,48,49,50,51,52]. Previous phytochemical studies have demonstrated moderate to high antioxidant activity, identifying the presence of various phenolic compounds, flavonoids, carboxylic acids and p-hydroxyacetophenone derivatives, diterpenes, and coumarins [51]. The presence of polyphenolic compounds, antioxidant activities, and traditional use against inflammatory conditions supports its potential neuroprotective effect. Nevertheless, the effect of *Pq* in a neurodegenerative context remains unexplored.

*Drosophila melanogaster* (hereafter *Drosophila* or fly) has been widely used as an in vivo platform for screening and rapid validating of neuroprotective compounds before mammalian assessment. Both genetic and toxin-induced PD fly models have clarified molecular mechanisms involved. Although flies lack a nigrostriatal pathway, certain arthropod brain regions functionally resemble mammalian Substantia Nigra and basal ganglia [53]. Notably, either genetic or toxin-induced PD fly models reproduce progressive dopaminergic neurodegeneration correlated with motor and non-motor features observed in PD patients [54,55,56]. Among genetic models, overexpression of the human α-syn gene (*hSNCA*), either its wild-type or pathogenic point-mutated version, has been achieved in flies [57,58]. A codon-optimized version of *hSNCA* (*SNCA.J*) increases expression levels of α-syn by 20-fold [59], mimicking locus multiplications in familial PD [60]. Expressing *hSNCA* codon optimized in fly dopaminergic neurons leads to the increase in ROS and RNS, decreased antioxidant activity by reduced glutathione (GSH), altered catalase and SOD activity [61], lipoperoxidation, ferroptosis, mitochondrial fragmentation [62], and synaptic disruption [59]. These findings highlight the use of this transgenic line for the search and first-line validation of neuroprotective compounds, as it reproduces key mechanisms of PD pathology.

This work aims to assess the biological activity of *P. quadrangularis* in a neurodegenerative context. A hydroalcoholic extract (HAE) and four subfractions of *Pq* were obtained. The in vitro antioxidant activity was assessed, and HAE exhibited a consistent response, being selected for further assays. The phytochemical composition of HAE-*Pq* was unveiled by UHPLC-MS, displaying high flavonoid content, along with coumarin, phenolic acids, and sixteen additional metabolites. Cell viability and the antioxidant capacity of HAE-*Pq* in SH-SY5Y cells were tested, resulting in the attenuation of ROS-induced levels by hydrogen peroxide (H_2_O_2_). Finally, in vivo assays were conducted using a *Drosophila* PD model induced by human α-syn overexpression in dopaminergic neurons. The supplementation of 1 mg/mL of HAE-*Pq* delayed the onset of motor impairments in female PD flies by one week. These findings highlight the value of *Drosophila* as first-line model for screening neuroprotective compounds and suggest the neuroprotective potential of *Pq*, warranting further investigations.

## 2. Materials and Methods

### 2.1. Bioethical and Safety Issues

All experimental procedures were approved by the Comité de Ética en Investigación Científica of the Universidad de Antofagasta (No. 417/2023; approved on 20 April 2023) and conducted in compliance with the guidelines of the Agencia Nacional de Investigación y Desarrollo (ANID). Experimental protocols involving *Drosophila melanogaster* were conducted in a laboratory approved by the Servicio Agrícola y Ganadero de Chile (SAG) (No. 5489/2023; approved on 29 August 2023).

### 2.2. Chemicals and Reagents

Ethanol (EtOH), n-hexane (n-hex), dichloromethane (DCM), ethyl acetate (EtOAc), methanol (MeOH), sodium carbonate (Na_2_CO_3_), gallic acid (GA), quercetin (QE), acetic acid (CH_3_COOH), and hydrochloric acid (HCl), ferric chloride hexahydrate (FeCl_3_·6H_2_O), 2,2-diphenyl-1-picrylhydrazyl (DPPH), 2,2′-azinobis(3-ethylbenzothiazoline-6-sulfonic acid) (ABTS), potassium persulfate (K_2_S_2_O_8_), ferrous chloride (FeCl_2_), dimethyl sulfoxide (DMSO) were purchased from Merck (Darmstadt, Germany); Folin–Ciocalteu reagent (F-C), anhydrous aluminum chloride (AlCl_3_), dibasic sodium phosphate dihydrate (Na_2_HPO_4_·2H_2_O), sodium nitrite (NaNO_2_), monobasic sodium phosphate monohydrate (NaH_2_PO_4_·H_2_O), ferrozine (FZ), propionic acid, methyl 4-hydroxybenzoate (Nipagin) were purchased from Sigma-Aldrich (St. Louis, MO, USA); molecular biology grade water (H_2_O-GBM) purchased from Corning (New York, NY, USA), trihydrate sodium acetate (CH_3_COONa·3H_2_O) purchased from Amresco (Solon, OH, USA), (2,4,6-tris(2-pyridyl)-s-triazine (TPTZ) purchased from TCI-Chemicals (Tokyo, Japan), (±)-6-hydroxy-2,5,7,8-tetramethylchroman-2-carboxylic acid (Trolox) purchased from Fluka (Dorset, UK), ethylenediaminetetraacetic acid (EDTA) purchased from VWR (VWR Life Science, Radnor, PA, USA), agar-agar purchased from TCL (Santiago, Chile), Mont Blanc baking powder-free flour, yeast (Lefersa), sucrose (Iansa), and commercial blue dye (Gourmet). Dulbecco’s Modified Eagle Medium (DMEM), phenol red-free DMEM, fetal bovine serum (FBS), penicillin, streptomycin, polyclonal rabbit (Rb) anti-TH antibody, goat anti-Rb Alexa Fluor™ 555, ProLong^TM^ Diamond Antifade Mountant, and 2′,7′-Dichlorofluorescin diacetate (DCFH-DA) were obtained from Thermo Fisher Scientific (Waltham, MA, USA). CellTiter 96^®^ AQueous One Solution Cell Proliferation Assay was acquired from Promega Corporation (Madison, WI, USA).

### 2.3. Botanical Material

The aerial parts (branches, leaves, and flowers) of *Parastrephia quadrangularis* (Meyen) Cabrera were collected in April 2022 near Quebrada de Chita, in the Antofagasta Region (Region II) of Chile. The collection site was located at −22°26′6.50″ S, −68°09′16.7″ W, and the altitude is 3925 m.a.s.l. (Figure 1a–d). Plant materials were dried in the dark at room temperature (RT), then finely ground using a mechanical mill and stored at RT until further use. The plant specimens were taxonomically identified and authenticated at the Department of Botany, University of Concepción, Chile, and a voucher specimen was deposited under the number M002-2022 in the herbarium of the laboratory of Química Biológica, Universidad de Antofagasta.

### 2.4. Preparation of the Hydroalcoholic Extract and Sub-Fractions

A hydroalcoholic extract of *Parastrephia quadrangularis* (HAE-*Pq*) was prepared by macerating approximately 1 kg of dried and ground plant material in a cloth bag with 2 L of a 1:1 ethanol:water (EtOH:H_2_O) mixture for 72 h at RT. The extract was then filtered through Whatman No. 4 filter paper and concentrated under reduced pressure using a rotary evaporator at 45 °C to one-quarter of its initial volume. This extraction process was repeated several times until the solution became colorless. The resulting concentrate was lyophilized and stored at 4 °C until further use. To reduce the chemical complexity of the extract, successive solvent partitioning was performed using solvents of increasing polarity. For this, 835 g of dried and ground plant material were macerated in a glass container with n-hexane for 24 h. The mixture was then filtered and evaporated to dryness using a rotary evaporator at 45 °C, yielding a gummy residue labeled n-hex. The same procedure was repeated sequentially with dichloromethane (DCM), ethyl acetate (EtOAc), and methanol (MeOH), yielding the corresponding DCM, EtOAc, and MeOH subfractions. All fractions were stored at 4 °C until further analysis.

### 2.5. UHPCL-ESI-QTOF-MS Analysis

The identification of secondary metabolites present in HAE-*Pq* was performed on a UHPLC-ESI-QTOF-MS system, including an Ultimate 3000 RS UHPLC system with Chromeleon 6.8 software (Dionex GmbH, Idstein, Germany) and a Bruker maXis ESI-QTOF-MS system with Data Analysis 4.0 software (Bruker Daltonik GmbH, Bremen, Germany). For analysis, 5 mg of HAE-*Pq* was dissolved in 500 µL of 80% methanol, filtered through a polytetrafluoroethylene (PTFE) membrane, and 3 µL of HAE-*Pq* was injected into the system. The chromatographic system included a quaternary pump, an autosampler, a thermostated column compartment, and a photodiode array detector. A binary gradient system was used for elution, with eluent (A) consisting of 0.1% formic acid in water and eluent (B) consisting of 0.1% formic acid in 90% acetonitrile. The gradient profile was as follows: isocratic 12% B (0–1 min), 12–99% B (2–14 min), isocratic 88% A (14–16 min). The separation was performed on a Kinetex C18 column (100 mm ø 2.1 mm) at a flow rate of 0.4 mL/min. ESI-QTOF-MS analyses were performed in negative and positive ion mode, with a scan range of 50–1300 *m*/*z*. Electrospray ionization (ESI) parameters included a capillary temperature of 230 °C, a capillary voltage of 4.5 kV, a dry gas flow rate of 8 L/min, and a nebulization pressure of 2.5 bar. The experiment was performed in automated MS/MS mode, and structural characterization of secondary metabolites was based on high-resolution comprehensive MS, fragmentation patterns, literature, and database comparisons.

### 2.6. Total Polyphenol Content

The total phenolic content (TPC) was determined using the Folin–Ciocalteu colorimetric method. The procedure was performed as described by Ainsworth et al. (2007) [63], with slight modifications. Briefly, 20 µL of extract (5 mg/mL) were mixed with 100 µL of 10% (*v*/*v*) Folin–Ciocalteu reagent and incubated for 5 min at RT. Then, 80 µL of sodium carbonate solution (700 mM) was added, followed by 1 min of stirring and a subsequent incubation for 60 min at RT. For the calibration curve, standard solutions of gallic acid ranging from 0 to 500 µg/mL were prepared. Absorbance was measured at 765 nm, and the results were expressed as milligrams of gallic acid equivalents per gram of extract (mg GAE/g extract). All assays were conducted in 96-well microplates and performed in triplicate. Absorbance was quantified by spectrophotometric analysis using a BioTek SYNERGY HTX multimode plate reader, equipped for absorbance, luminescence, and fluorescence detection. All data was obtained using Gen 5.0 software.

### 2.7. Total Flavonoid Content

The total flavonoid content (TFC) was determined using the aluminum chloride colorimetric method. The procedure was based on the protocol described by Shao et al. (2022) [64], with slight modifications. Briefly, 20 µL of extract (5.5 mg/mL) were mixed with 140 µL of DMSO and 30 µL of 5% sodium nitrite, and the mixture was allowed to stand for 5 min at RT. Then, 30 µL of 10% aluminum chloride were added, followed by a 15 min incubation at RT. For the calibration curve, quercetin standard solutions ranging from 0 to 250 µg/mL were prepared. Absorbance was measured at 510 nm, and the results were expressed as milligrams of quercetin equivalents per gram of extract (mg QE/g extract).

### 2.8. In Vitro Antioxidant Activity

#### 2.8.1. Ferric Reducing Antioxidant Power

The ferric reducing antioxidant power (FRAP) was determined using the colorimetric method based on the reduction of Fe^3+^ to Fe^2+^. The procedure followed the protocol described by Akter et al. (2016) [65], with slight modifications. The FRAP reagent was freshly prepared by mixing acetate buffer (300 mM, pH 3.6), TPTZ solution (10 mM dissolved in 40 mM HCl), and FeCl_3_·6H_2_O solution (20 mM dissolved in 40 mM HCl) in a 10:1:1 (*v*/*v*/*v*) ratio. A total of 30 µL of extract (667 µg/mL) was mixed with 170 µL of freshly prepared FRAP reagent, followed by shaking for 1 min and incubation at 37 °C for 30 min. For the calibration curve, Trolox standard solutions ranging from 0 to 15.6 µg/mL were used. Absorbance was measured at 593 nm, and results were expressed as milligrams of Trolox equivalents per gram of extract (mg TE/g extract).

#### 2.8.2. Determination of Metal Chelating Activity

The ferrous ion chelating activity was determined using a colorimetric method based on the ability of antioxidants to chelate Fe^2+^ ions. The procedure was performed as previously described by Aktumsek et al. (2013) [66], with slight modifications. Briefly, 50 µL of extract (0–1000 µg/mL, logarithmic concentrations) were mixed with 10 µL of FeCl_2_ solution (2 mM). Then, 20 µL of ferrozine solution (5 mM) and 70 µL of MeOH were added, followed by a 10 min incubation at RT. EDTA standard solutions (0–1000 µg/mL) were used to construct the calibration curve. Absorbance was measured at 562 nm. Chelating activity was expressed as the percentage of Fe^2+^ ions bound, calculated according to Equation (1). The IC_50_ value, representing the concentration of extract or standard required to chelate 50% of Fe^2+^ ions in solution, was determined using GraphPad Prism version 10.5.0.

Inhibition (%) = [(Abs*_control_* − Abs*_sample_*)/Abs*_control_*] × 100 (1)

#### 2.8.3. Determination of DPPH Radical-Scavenging Activity

Antioxidant activity was assessed using a colorimetric method based on the scavenging of the DPPH^•^ radical. The procedure followed the protocol described by Akter et al. (2016) [65], with slight modifications. Briefly, 70 µL of DPPH solution (0.2 mM) was mixed with 30 µL of extract (0–1000 µg/mL, logarithmic concentrations), followed by a 30 min incubation at RT. The calibration curve was prepared using Trolox standard solutions ranging from 0 to 1000 µg/mL. Absorbance was measured at 517 nm. The blank consisted of the MeOH and DPPH^•^ solution. Results were expressed as the percentage of DPPH^•^ inhibition, and the IC_50_ value was calculated. The IC_50_ corresponds to the concentration of standard or extract required to inhibit 50% of the DPPH^•^ radical. Both parameters were calculated using the same procedure described for chelating activity (Equation (1)).

#### 2.8.4. Determination of ABTS Radical-Scavenging Activity Material

Antioxidant activity was assessed using a colorimetric method based on the scavenging of the ABTS^•+^ radical. The procedure was based on the protocol described by Bekir et al. (2013) [67], with slight modifications. The ABTS^•+^ radical was generated by mixing a 7 mM ABTS solution with 2.5 mM of K_2_S_2_O_8_ in 100 mM phosphate-buffered saline (PBS, pH 7.4), and the mixture was incubated in darkness at RT for 16 h. Prior to use, the radical solution was diluted with PBS to obtain an absorbance of 0.700 ± 0.02 at 734 nm. Then, 30 µL of extract (0–1000 µg/mL, logarithmic concentrations) were mixed with 170 µL of the diluted ABTS^•+^ solution and allowed to react for 6 min at RT. For the calibration curve, Trolox standard solutions ranging from 0 to 1000 µg/mL were used. Absorbance was measured at 734 nm. The blank consisted of PBS and an ABTS^•+^ solution. Results were expressed as the percentage of ABTS^•+^ inhibition, and the IC_50_ value was calculated. The IC_50_ corresponds to the concentration of an extract or standard required to inhibit 50% of ABTS^•+^ radicals. Both parameters were calculated using the same procedure for metal chelating activity (Equation (1)).

### 2.9. Cell Culture

Human neuroblastoma SH-SY5Y cell line was obtained from the Neurobiology Laboratory (Universidad de Antofagasta, Antofagasta, Chile), kindly provided by Dr. María E. Andrés (Pontificia Universidad Católica de Chile, Santiago, Chile). SH-SY5Y cells were cultured in Dulbecco’s Modified Eagle Medium supplemented with 10% fetal bovine serum, 100 U penicillin, and 100 mg/mL streptomycin. Cells were maintained in an incubator with 5% CO_2_ at 37 °C.

### 2.10. Cell Viability

Cell viability was assessed in SH-SY5Y cells exposed to HAE-*Pq*. Briefly, SH-SY5Y cells were seeded into 96-well plates at 30,000 cells/well in DMEM supplemented with 10% FBS. A stock solution of HAE-*Pq* (10 mg/mL) was prepared by resuspending the lyophilized extract in cell culture grade ultra-pure water and filtering through a 0.22 μm membrane. After 24 h, the medium with increasing concentrations of HAE-*Pq* (0–400 µg/mL) in phenol red-free DMEM supplemented with 1% FBS was added to cells and incubated for 24 h at 37 °C. Cell viability was determined using the CellTiter 96^®^ AQueous One Solution Cell Proliferation Assay, a colorimetric assay in which the reduction of the yellow tetrazolium salt to a soluble formazan product is directly proportional to the number of metabolically active cells. For the assay, 20 µL of MTS reagent was added to each well and incubated for 3 h at 37 °C. Absorbance was measured at 490 nm using a microplate reader. Results were expressed as the percentage of cell viability relative to the untreated control (% to control).

### 2.11. Measurement of Intracellular ROS Levels

SH-SY5Y cells (30,000 per well) were seeded in 96-well microplates and allowed to adhere for 24 h in DMEM supplemented with 10% FBS. After incubation, the medium was replaced with phenol red-free DMEM without FBS containing 40 μM 2′,7′-dichlorodihydrofluorescein diacetate (DCFH-DA), and cells were incubated for 30 min at 37 °C in the dark, as previously described [68]. Basal ROS levels were immediately measured. For experiments involving HAE-*Pq* and H_2_O_2_, cells were pre-treated with 200 μg/mL of HAE-*Pq* for 16 h. Subsequently, the medium was replaced with phenol red-free DMEM without FBS containing 40 μM DCFH-DA, and cells were incubated for a further 30 min at 37 °C in the dark. After DCFH-DA loading and washing with PBS, increasing concentrations (0–500 μM) of hydrogen peroxide (H_2_O_2_) were added, and cells were incubated for an additional 3 h. Fluorescence intensity was measured using a SYNERGY-HT microplate reader (BioTek, Winooski, VT, USA) with an excitation wavelength of 485/20 nm and emission at 528/20 nm. Results were expressed as a percentage change in fluorescence intensity relative to the control group.

### 2.12. Drosophila Melanogaster Stocks and Husbandry

All fly stocks were reared at 19 °C under a 12/12 h light/dark cycle with 50–60% relative humidity (RH) and maintained on a standard fly food diet as previously described [69]. For experimental procedures, flies were raised at 25 °C. The following strains were used and obtained from Bloomington *Drosophila* Stock Center (Department of biology, Indiana University, Bloomington, IN, USA): *w^1118^* (#5905), *w^1118^*; *P{w[+mC] = UAS-SNCA.J}7/TM3*, *Sb^1^* (#51376), and w[*]; *P{w[+mC] = th-GAL4.F}3* (#8848). Control and PD flies were generated through genetic crosses using the UAS/GAL4 binary system [70]. Control flies (hereafter CTRL or *th* > +) were obtained by crossing *w^1118^* and *th-GAL4* (III), while PD flies (hereafter PD or *th* > *SNCA.J*) were generated by crossing *UAS-SNCA.J/TM3* with *th-GAL4* (III).

### 2.13. Drosophila Exposure to HAE-Pq

HAE-*Pq* was supplemented with standard fly food during larvae and adult stages. Briefly, the lyophilized HAE-*Pq* was resuspended in autoclaved distilled water at a final concentration of 10 mg/mL. A volume of this stock solution was added to freshly prepared fly food before solidification to achieve a final concentration of 0, 0.1, and 1 mg/mL of HAE-*Pq*. The following day, adult control or PD flies were transferred to vials supplemented with HAE-*Pq* and subsequently transferred to fresh vials every 2–3 days until the day of behavioral analysis. For larvae exposure, parental flies were allowed to mate and lay eggs in vials supplemented with HAE-*Pq*.

### 2.14. Food Intake: Modified Capillary Feeding (CAFE) Assay

Food intake in flies was evaluated using a modified version of CAFE assay, as previously described by Segu et al. (2023), with minor modifications [71]. The modified CAFE assay quantifies food consumption by measuring the reduction in liquid food delivered in a microtip. The consumed volume was calculated by applying the formula for the volume of a truncated cone, using the initial (h_1_) and final (h_2_) heights of the liquid column. To apply this formula, it was necessary to determine the radius at h_1_ and h_2_. For this purpose, images of the inner radius of 10 microtips were captured at the base (h_1_) and at the meniscus level corresponding to 10 µL (h_2_), using an Olympus CX31 microscope equipped with a Swift 1.3-megapixel camera (Figure S1a–d). Images were analyzed using Fiji (ImageJ2, version 2.16.0/1.54p; National Institutes of Health, Bethesda, MD, USA), and the radius was measured with a calibration scale bar. The mean values for r_1_ and r_2_ were obtained. The equation of the line was used to obtain the slope (*m*), which was then used to calculate the radius (r_3_) at the final height (h_3_) corresponding to the meniscus level at the end of the assay (Figure S1e). The r_3_ value was then incorporated into the truncated cone equation to determine the final volume remaining in the microtip (Figure S1f).

Flies from different vials were randomly allocated into experimental groups, which consisted of control or PD flies exposed to vehicle (water; 0 mg/mL HAE-*Pq*) or different concentrations of HAE-*Pq* (0.1 or 1 mg/mL). The experimental unit was defined as a group of 20 flies (10 males and 10 females), either control or PD. For each condition, five experimental units (*n* = 5 tubes, 20 flies each) were analyzed, corresponding to a total of 100 flies per condition. In total, 30 experimental units were analyzed, corresponding to 600 flies across all conditions. The day before the assay, flies were selected under ice anesthesia and transferred to fresh vials. On the day of the assay, flies were transferred into 50 mL conical tubes containing 15 mL of 1% agar and incubated for 1 h at 25 °C and 50–60% HR in darkness. Afterward, two microtips containing 10 µL liquid food (0.25 M sucrose plus 1:1000 blue commercial food dye) were inserted into each tube (Figure S1g). Liquid food was supplemented with 0, 0.1 or 1 mg/mL HAE-*Pq*. An evaporation control was included in experimental groups, which consisted of an identical setup without individuals. Flies were allowed to feed for 4 h at 25 °C, 50–60% HR in darkness. The modified CAFE assay was conducted during the Zeitgeber time (ZT) 01–05. To obtain the initial and final heights, a calibrator vernier was used. The food intake was calculated with the formula presented in Equation (2):

V_consumed_ = V_initial_ − V_final_ − V_evapored_ (2)

Results were expressed as µL of food intake per fly. No flies were excluded in this analysis.

### 2.15. Viability in Drosophila: From Embryo to Adult

Viability in control and PD flies was evaluated by monitoring developmental progression from embryo to adult stage to assess the effect of HAE-*Pq* during the larval stage. This assay was adapted from Liu et al. (2019) [72], with some modifications. The experimental groups in this assay consisted of control or PD flies randomly allocated and exposed to vehicle (water; 0 mg/mL HAE-*Pq*) or to different concentrations of HAE-*Pq* (0.1 or 1 mg/mL). For each condition, the experimental unit consisted of one vial containing 30 parental flies (15 males and 15 females). Three experimental units (*n* = 3 vials, 30 flies each) were established per condition for both control and PD genotypes exposed to 0.1 or 1 mg/mL HAE-*Pq*, while four experimental units (*n* = 4) were used for control and PD flies exposed to 0 mg/mL HAE-*Pq*. Parental flies used to generate control and PD genotypes were allowed to mate and lay eggs in vials containing standard food supplemented with HAE-*Pq* (0, 0.1, or 1 mg/mL) for 24 h at 25 °C. Afterwards, the parentals were removed, and the embryos were left to develop until adult emergence. The number of emerging adult flies was recorded every 24 h. The number of emerged adult flies in the 0 mg/mL HAE-*Pq* group per genotype was set as 100%, and emergence in 0.1 and 1 mg/mL conditions was normalized to this control (0 mg/mL HAE-*Pq*). No flies were excluded in this analysis.

### 2.16. Viability in Adult Flies

Adult viability of control and PD flies was assessed following a previously published protocol by Poleto et al. (2024) [73], with minor modifications. The experimental groups consisted of control or PD flies randomly allocated and exposed to vehicle (water; 0 mg/mL HAE-*Pq*) or to different concentrations of HAE-*Pq* (0.1 or 1 mg/mL). For each condition, the experimental unit consisted of a vial containing 20 flies (10 males and 10 females) aged 1–3 days post-eclosion. Five experimental units (*n* = 5 vials, 20 flies each) were established per condition for both control and PD genotypes exposed to 0, 0.1, or 1 mg/mL HAE-*Pq*. In total, 30 experimental units were analyzed, corresponding to 600 flies (100 flies per condition). To start the experiment, flies were transferred to vials containing standard fly food supplemented with HAE-*Pq* (0, 0.1, or 1 mg/mL). Flies were maintained with a supplemented diet for 7 days and were transferred to fresh vials every 24 h, at which time the number of deaths was recorded. The number of surviving adult flies in the 0 mg/mL HAE-*Pq* condition per genotype was set as 100%, and survival in the 0.1 and 1 mg/mL conditions was normalized to this control (0 mg/mL). Results are expressed as the percentage of total surviving flies. No flies were excluded in this analysis.

### 2.17. Motor Performance: Climbing Assay

The motor response of control and PD flies, both exposed to vehicle (0 mg/mL HAE-*Pq*) or to 1 mg/mL HAE-*Pq*, was assessed using the startle-induced negative geotaxis response (also known as the climbing assay), which measures the ability of flies to climb above a predefined threshold within a given time. This assay was performed as previously described by Narwal et al. (2024), with slight modifications [62]. Flies from different vials were randomly allocated to experimental groups. For each condition, the experimental unit consisted of 10 female flies placed in a vertical cylindrical polystyrene tube (*n* = 10 tubes, 10 flies each). Ten experimental units were analyzed per condition, corresponding to 100 flies per condition. In total 400 flies were used across all conditions. On the day of the assay, flies were anesthetized on ice and transferred into polystyrene tubes. After 45 min of acclimation at RT, flies were gently tapped down to the bottom of the tube and allowed to climb for 10 s. This procedure was repeated 10 times with a 1 min rest interval between trials. The number of flies that climbed above 8 cm within the 10 s was recorded. Assays were conducted between 01 and 04 ZT. Results are presented as the percentage of flies that escaped the threshold. No flies were excluded in this analysis.

### 2.18. Immunofluorescence

An immunofluorescence against tyrosine hydroxylase (TH) was performed to identify dopaminergic neurons in fly brains. Polyclonal rabbit (Rb) anti-TH antibody (AB152, Chemicon^®^; Temecula, CA, USA) was used to stain whole brains following previously published protocols, with minor modifications [69,74]. Anti-TH (1:250) was incubated with whole brains for 3 nights at 4 °C with agitation, followed by 3 washes with PBT (PBS 1×+ 0.3% Triton X-100), each one for 15 min. Goat anti-Rb Alexa Fluor^TM^ 555 (1:200; A21428, Thermo Fisher Scientific, Waltham, MA, USA) secondary antibody was incubated for 3 h at RT with agitation. Brains were again washed 3 times with PBT and stored in PBS 1× at 4 °C until mounting with ProLong^TM^ Diamond Antifade Mountant solution (P36966, Thermo Fisher Scientific, Waltham, MA, USA).

### 2.19. Imaging and Quantification of TH-Positive Cells

Images from whole adult fly brains stained with anti-TH were acquired in a Leica TCS SP8 confocal microscope (Leica Microsystems, Wetzlar, Germany), at 1024 × 1024 pixels resolution using LAS X (Leica Application Suite X) software, version 1.1.0.12420. Anti-Rb Alexa Fluor 555 was excited at 552 nm and detected at 560–610 nm. Images were processed in Fiji, version 1.54p (NIH, Bethesda, MD, USA), with LUT gray. Quantification of dopaminergic neurons was performed as previously described by Zárate et al. (2022) [69], identifying the soma of each cluster (PPM1/2, PPM3, PPL1, PPL2ab, and PPL2c) in the right and left hemispheres along the Z-stack. The somas were counted manually through the Z-stack using the multi-point selection tool in Fiji. The experimental groups in this assay were allocated randomly and consisted of control and PD flies, exposed either to vehicle (0 mg/mL HAE-*Pq*) or to 1 mg/mL HAE-*Pq*. The experimental unit was defined as a whole brain. Approximately 10–15 brains were employed per condition, for both control and PD genotypes exposed to 0 or 1 mg/mL HAE-*Pq*, corresponding to a total of 40–60 hemispheres across all experimental groups. Hemispheres in which the integrity of the tissue was compromised due to dissection were excluded from the analysis. The Z-projection function with the maximum intensity option was used to obtain a representative image of brains and to verify the location and number of somas.

### 2.20. Statistical Analysis

All data obtained from the experiments are expressed as the mean ± Standard Error of the Mean (SEM) or the interquartile range with maximum and minimum ranges. The Shapiro–Wilk test was applied to assess normality and determine whether parametric or non–parametric statistical tests were appropriate. For data meeting parametric assumptions, one– or two–way analysis of variance (ANOVA) followed by the Bonferroni or Tukey post hoc test was applied. For data not meeting these assumptions, the Kruskal-Walli’s test followed by Dunn’s post hoc test was used to compare data and one factor. GraphPad Prism software, version 10.5.0 (GraphPad Software, Inc., La Jolla, CA, USA), was used to perform statistical analysis. Statistical significance was set at * *p* < 0.05, ** *p* < 0.01, and *** *p* < 0.001. The number of experimental units and the specific tests for statistical analysis of each experiment are indicated in figure legends.

## 3. Results

### 3.1. Phenol and Flavonoid Content of P. quadrangularis

**Figure 2:**
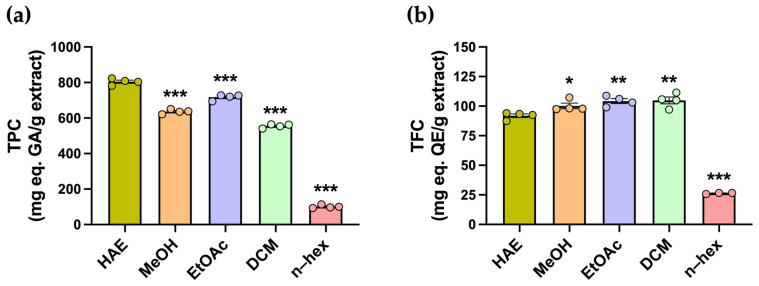
Estimation of the total phenolic and flavonoid content of *P. quadrangularis*. (**a**) Total phenolic content (TPC) was measured in the hydroalcoholic extract (HAE, olive green), methanolic (MeOH, light orange), ethyl acetate (EtOAc, light violet), dichloromethane (DCM, light green), and n-hexane (n-hex, light pink) subfractions of *P. quadrangularis*. The data are presented as the mean ± SEM of the mg equivalent of gallic acid (AG) per gram of extract. (**b**) Total flavonoid content (TFC) was measured in the HAE and subfractions of *P. quadrangularis*. The data are presented as the mean ± SEM of the mg equivalent of quercetin (QE) per gram of extract. Statistical analyses were conducted using one–way ANOVA, followed by a Bonferroni post hoc test (*n* = 4), comparing each subfraction with the HAE. * *p* < 0.05, ** *p* < 0.01, and *** *p* < 0.001.

Polyphenolic compounds comprise a diverse group of secondary metabolites with antioxidant properties, including flavonoids. The total polyphenol content (TPC) and total flavonoid content (TFC) were determined using colorimetric assays (Figure 2a). The highest TPC was observed in the HAE, with 804.4 ± 8.6 mg GAE/g, whereas all subfractions exhibited significantly lower values (*** *p* < 0.001) (Table 1, Figure 2a). In contrast, the highest TFC values were obtained for the DCM, EtOAc, and MeOH subfractions, with 104.8 ± 3.0, 104.3 ± 2.1, and 100.2 ± 2.4 mg QE/g, respectively, while the HAE presented a slightly but significantly lower TFC of 91.9 ± 1.5 mg QE/g (Table 1, Figure 2b).

### 3.2. In Vitro Antioxidant Activity of P. quadrangularis

**Figure 3:**
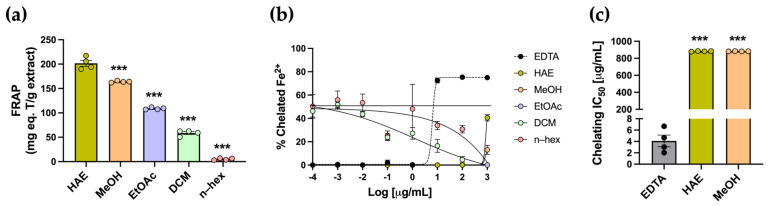
Determination of the ferric reducing antioxidant power and metal chelating activity of *P. quadrangularis* extracts. (**a**) Ferric reducing antioxidant power (FRAP) was measured in the HAE (olive green) and subfractions (MeOH, light orange; EtOAc, light violet; DCM, light green, n–hex, light pink) of *P. quadrangularis*. Data is expressed as the mean ± SEM of the mg equivalent of Trolox (T) per gram of extract. Analysis was performed using one-way ANOVA followed by a Bonferroni post hoc test (*n* = 4), comparing each subfraction with the HAE. *** *p* < 0.001. Metal chelating activity was measured in the HAE and subfractions of *P. quadrangularis*. (**b**) Inhibition curves of Fe^2+^ chelation for HAE, MeOH, EtOAc, DCM, and n–hex are presented. The inhibition curve of EDTA (black) was used as the standard. The data are presented as the mean ± SEM of the % of chelated Fe^2+^. (**c**) IC_50_ values of HAE, MeOH, and EDTA (gray) are presented in the graph. The data are expressed as the mean ± SEM of the concentration (µg/mL) that produces 50% chelation of Fe^2+^. The statistical analysis was performed using one–way ANOVA, followed by a Bonferroni post hoc test (*n* = 4), comparing each extract’s IC_50_ value with the EDTA standard. *** *p* < 0.001.

Antioxidants can neutralize free radicals through different mechanisms, including electron transfer, metal chelation, and hydrogen atom transfer. The FRAP colorimetric assay was used to evaluate the reduction of Fe^3+^ to Fe^2+^ ions. The highest FRAP value was observed in the HAE, with 201.6 ± 6.1 mg TE/g, whereas the subfractions exhibited significantly lower values (*** *p* < 0.001) (Table 1, Figure 3a). The chelating activity assay was conducted to assess the ability of the extract and subfractions to capture Fe^2+^ ions. All metal–chelating curves, including HAE, subfractions, and EDTA, are presented in Figure 3b. As expected, the standard EDTA exhibited the typical sigmoidal response, with an IC_50_ of 4.661 ± 1.02 µg/mL (Figure 3c). Both HAE and subfractions did not exhibit the typical sigmoidal curve and displayed low chelating activity across the tested concentrations. IC_50_ values were obtained only for the HAE and MeOH subfractions, both of which were significantly higher than the EDTA standard, indicating lower chelating potency (*** *p* < 0.001) (Table 1, Figure 3c).

**Figure 4:**
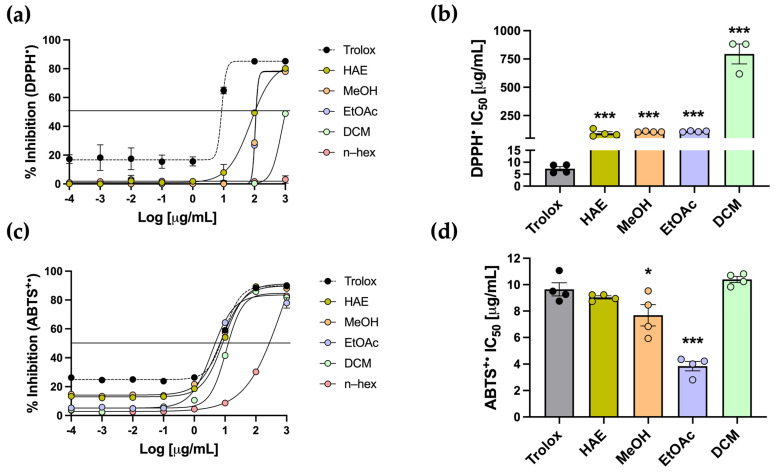
Determination of DPPH and ABTS^•+^ inhibition of *P. quadrangularis*. (**a**) The inhibition curves of HAE (olive green), MeOH (light orange), EtOAc (light violet), DCM (light green), and n–hex (light pink) were compared with the Trolox standard (black) for DPPH, expressed as mean ± SEM of the % inhibition for DPPH. (**b**) IC_50_ values of HAE, MeOH, EtOAc, and DCM are shown relative to Trolox, expressed as the mean ± SEM of the concentration (µg/mL) required to inhibit 50% of each radical. (**c**) The inhibition curves and IC_50_ values (**d**) of *P. quadrangularis* extracts were compared with Trolox for the ABTS^•+^ radical. Statistical analyses for the IC_50_ values were performed using one–way ANOVA, followed by a Bonferroni post hoc test (*n* = 4), comparing each extract with the Trolox standard. * *p* < 0.05 and *** *p* < 0.001.

Another approach to evaluating antioxidant capacity is through radical scavenging assays, using DPPH and ABTS as radical species. Trolox was used as the standard antioxidant for both assays. In the DPPH assay, which is based on single–electron transfer neutralization, Trolox displayed the expected sigmoidal curve, recording an IC_50_ of 7.30 ± 0.89 µg/mL (Figure 4a,b). HAE-*Pq* and the subfractions exhibited dose–dependent activity, except for the n–hex subfraction, with no inhibition (Figure 4a). The IC_50_ values for HAE, MeOH, EtOAc, and DCM subfractions were all significantly higher than Trolox (*** *p* < 0.001) (Table 1, Figure 4b). Among the extracts, the DCM subfraction displayed the weakest activity, recording the highest IC_50_ value, while HAE, MeOH, and EtOAc displayed comparable values, which were lower than that of DCM (Table 1, Figure 4b).

The ABTS assay is based on hydrogen atom transfer and is carried out under physiological pH conditions. In this assay, Trolox exhibited the expected sigmoidal curve with an IC_50_ of 9.65 ± 0.50 µg/mL. The HAE and subfractions also showed a dose-dependent radical scavenging activity against ABTS^•+^ radicals (Figure 4c). Notably, the HAE, MeOH, EtOAc, and DCM exhibited sigmoidal curves comparable to that of the standard. Regarding IC_50_, the HAE and the DCM subfraction did not differ significantly from Trolox, indicating a scavenging capacity similar to the standard. The MeOH and EtOAc subfractions showed significantly lower IC_50_ values than the standard (* *p* < 0.05 and *** *p* < 0.001, respectively) (Table 1, Figure 4d), indicating an even greater scavenging capacity than Trolox.

Table 1 summarizes the results of in vitro antioxidant assays. Overall, the HAE-*Pq* demonstrated a consistent performance, particularly reflected in TPC and FRAP assays, although some subfractions showed comparable activity in specific tests. The metal-chelating assay showed that neither the HAE nor the subfractions displayed significant chelating power. In contrast, the ABTS scavenging assay showed that both the HAE and subfractions exhibited a similar inhibitory capacity as the antioxidant standard Trolox. Thus, based on the overall performance and consistent in vitro response, the HAE-*Pq* was selected for the subsequent analysis as a first approach.

### 3.3. UHPLC-ESI-QTOF-MS Analysis of HAE-Pq

**Figure 5:**
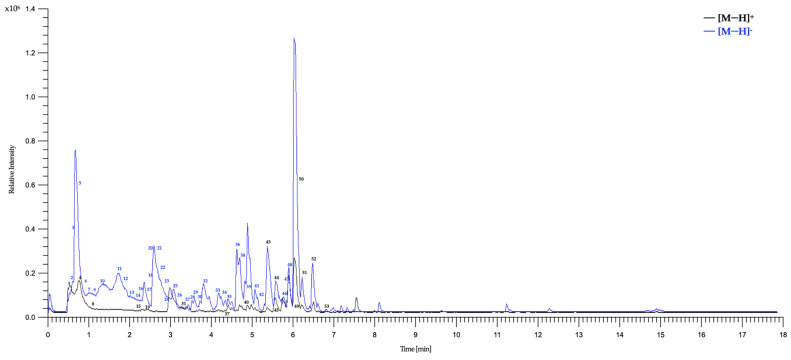
Total ion chromatograms (TIC) of the HAE-*Pq* using UHPLC-ESI-QTOF-MS in positive ionization [M−H]^+^ and negative ionization [M−H]^−^ modes. The numbers represent the tentatively identified compounds listed in Table 2.

To further characterize the phytochemistry of HAE-*Pq* and to identify the metabolites potentially associated with its antioxidant activity, we performed an UHPLC–ESI–QTOF–MS analysis. Fifty-three metabolites were identified and summarized in Table 2, including retention time, experimental *m*/*z* ratio, and relative abundance. The identified compounds mostly belong to different chemical families, including twenty-two flavonoids (peaks 27 to 29, 31 to 34, 36 to 39, 41 to 44, 46 to 50, 52, and 53), six flavonoid glycosides (peaks 12, 14, 17, 18, 22, and 24), six coumarins (peaks 11, 10, 15, 19, 26, and 45), three coumarin glycosides (peaks 1, 8, and 9), seven phenolic acids (peaks 2, 7, 16, 21, 23, 25, and 30), two carboxylic acids (peaks 3 and 6), one amino acid (peak 4), one hydroxybenzaldehyde (peak 5), one phenylpropanoid (peak 13), one fatty acid (peak 20), one aurone (peak 35), one chromene (peak 40), and one tremetone (peak 51) in the total ion chromatogram of the HAE-*Pq* (Figure 5).

**Figure 6:**
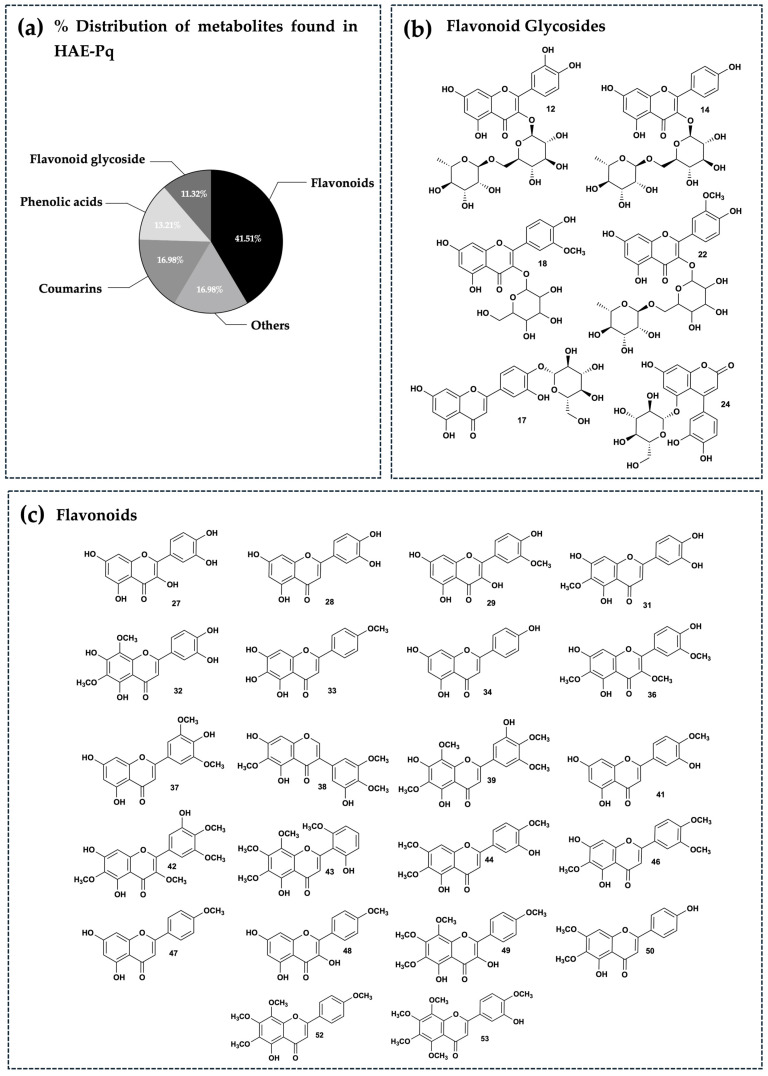
Phytochemical composition of HAE-*Pq*. (**a**) Percentage distribution of the fifty–three secondary metabolites found in UHPLC–ESI–QTOF–MS analysis and grouped according to their main chemical families. Chemical structures of the flavonoids identified in the extract. These have been separated into flavonoid glycosides (**b**) and flavonoids (**c**). The chemical structures of the other remaining metabolites identified can be seen in Figure S2a–c.

The distribution of secondary metabolites identified in HAE-*Pq* corresponds to flavonoids (glycosylated and non-glycosylated), which represent 52.83% of abundance (Figure 6a); this is the most abundant group of metabolites present in the extract (Figure 6b,c). The second most abundant family of compounds in the extract corresponds to coumarins (16.98%), followed by phenolic acids (13.21%), and the remaining 16.98% corresponds to metabolites from other chemical families, such as carboxylic acids, amino acids, hydroxybenzaldehydes, phenylpropanoids, fatty acids, aurones, and chromenes. The percentage distribution of metabolites is presented in Figure 6a. The structures of compounds are exhibited in Figure 6b,c and Figure S2a–c.

### 3.4. HAE-Pq Attenuates H_2_O_2_-Induced ROS Increase in SH-SH5Y

**Figure 7:**
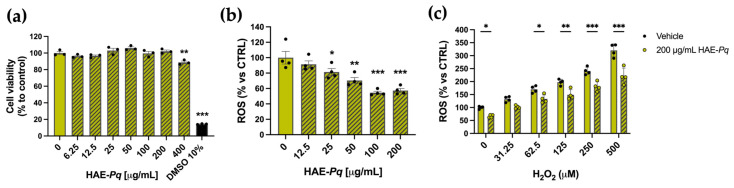
HAE-*Pq* exhibits no cytotoxic effects on SH-SY5Y cells and attenuates reactive oxygen species (ROS) production. (**a**) SH-SY5Y cell cultures were exposed to different increasing concentrations (0–400 μg/mL) of HAE-*Pq* for 24 h. DMSO (10%) was used as damage control. MTS reagent was added for 3 h to assess metabolic status and cell viability (*n* = 3). (**b**) SH-SY5Y cell cultures were exposed to increasing concentrations of HAE-*Pq* (0–200 μg/mL) for 16 h, and ROS production was measured with DCFH-DA (*n* = 4). (**c**) SH-SY5Y cells were pretreated with 200 µg/mL HAE-*Pq* or vehicle for 16 h and then exposed to different increasing concentrations (0–500 µM) of H_2_O_2_ for 3 h. ROS production was quantified using DCFH-DA (*n* = 4). Data are mean ± SEM. One–way ANOVA followed by the Bonferroni post hoc test was applied in (**a**,**b**), as compared to control (0 mg/mL HAE-*Pq*). Two–way ANOVA followed by Bonferroni post hoc test was used in (**c**), comparing H_2_O_2_ concentrations to control (0 μM H_2_O_2_ and 0 μg/mL HAE-*Pq*), and vehicle versus HAE-*Pq* treatments. * *p* < 0.05, ** *p* < 0.01, *** *p* < 0.001.

As the biological activity of HAE-*Pq* has not yet been assessed in neuronal contexts, the first biological test was conducted in SH-SY5Y cells to evaluate whether the extract exhibits a cytotoxic activity in a cell line with neuronal phenotype. Cells were exposed for 24 h to increasing concentrations (0–400 mg/mL) of HAE-*Pq*, and cell viability was assessed using the MTS assay. No cytotoxic effects were observed at the tested concentrations (Figure 7a). A slight decrease in cell viability was detected at the highest concentration (400 mg/mL), where cells exhibited 88.79 ± 1.59% survival (*p* = 0.001). A group of cells was exposed to 10% DMSO as damage control, which exhibited 14.40 ± 0.28% (*p* < 0.001).

As UHPLC-MS analysis unveiled a high content of flavonoids in the HAE-*Pq*, and due to the significant in vitro antioxidant activity, its effects on basal ROS production were assessed in SH-SY5Y. After 16 h of exposure, HAE-*Pq* reduced the basal ROS level in SH-SH5Y cells in a dose-dependent manner. The concentrations of 100 and 200 µg/mL resulted in a 54.56 ± 1.87% and 57.38 ± 2.63% reduction in ROS production, respectively, compared with the control group (Figure 7b; *p* < 0.001). Furthermore, to verify the antioxidant capacity of HAE-*Pq*, cells were exposed to a ROS inducer, H_2_O_2_. The increasing concentrations of H_2_O_2_ (from 31.25 to 500 µM) triggered the expected significant increase in ROS levels compared to the control group. Interestingly, a biocompatible dose of HAE-*Pq* (200 µg/mL) pretreatment attenuated the ROS increase after 3 h of exposure to H_2_O_2_ (Figure 7c). These results indicate that HAE-*Pq* does not exert cytotoxic effects, and its pre-incubation at 200 mg/mL for 16 h attenuates the increase in ROS levels by H_2_O_2_ exposition in a neuronal cell line. This antioxidant and protective effect supports its suitability for further neuroprotection assessment in in vivo assays using a neurodegenerative model of PD in *Drosophila*.

### 3.5. HAE-Pq Exhibits No Toxic Effects in Drosophila at Both Larval and Adult Stages

Since *Pq* exhibits a characteristic aroma, the first in vivo assay consisted of verifying whether flies consume standard fly food supplemented with HAE-*Pq*. Thus, for this purpose, the food intake rate was measured in control and PD flies using a modified version of the CAFE assay. Flies were exposed to liquid fly food supplemented with 0, 0.1, and 1 mg/mL of HAE-*Pq*. No significant differences in food intake were observed in control flies exposed to 0.1 (*p* = 0.14) or 1 mg/mL (*p* = 0.22) HAE-*Pq* compared to age-matched non-exposed individuals. Similar results were observed in PD flies, where HAE-*Pq* did not change food intake at 0.1 (*p* = 0.11) or 1 mg/mL (*p* = 0.41) (Figure S3). When comparing intake between genotypes, PD flies exhibited significantly higher food consumption than control flies at 0.1 mg/mL HAE-*Pq* (*p* < 0.001). This difference was not observed at 0 (*p* = 0.44) or 1 mg/mL (*p* = 0.25) (Figure S3). These results indicate that HAE-*Pq* extract does not induce aversive effects in flies, confirming that flies consume the extract.

**Figure 8:**
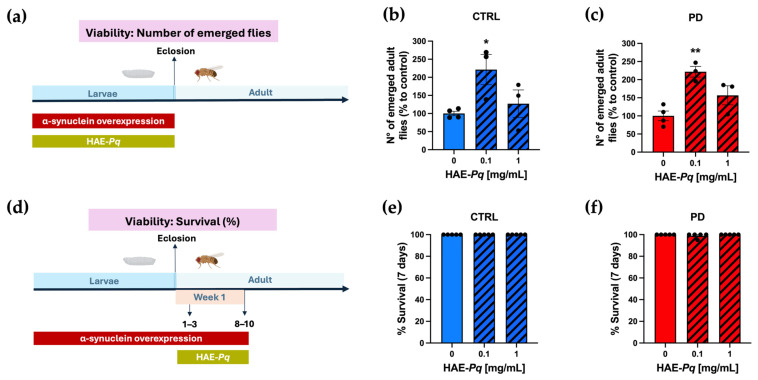
HAE-*Pq* exhibits no toxic effects on larvae and adult flies. (**a**) Schematic representation of the experimental design for larval viability assessment. Parental flies to generate control (CTRL, blue) and PD (red) genotypes were crossed on a standard fly diet supplemented with increasing concentrations of HAE-*Pq* (0, 0.1, 1 mg/mL) for 24 h at 25 °C. Larvae were grown on a standard fly diet (0 mg/mL, solid bars) or supplemented with HAE-*Pq* (0.1 and 1 mg/mL, hatched bars), and the number of emerged adult control (**b**) and PD (**c**) flies was recorded during 24 h for 4 days (*n* = 3). Data is presented as the mean ± SEM of total emerged flies. One–way ANOVA was performed, followed by the Bonferroni post hoc test, comparing different concentrations vs. 0 mg/mL HAE-*Pq*. * *p* < 0.05 and ** *p* < 0.01. (**d**) Schematic representation of the experimental design for adult viability assessment. Adult flies of 1–3 days old were exposed to a standard diet supplemented with increasing concentrations of HAE-*Pq* (0, 0.1, 1 mg/mL) for 7 days at 25 °C. Deaths were recorded every 24 h (*n* = 5). The data are presented as the mean ± SEM of control (**e**) or PD (**f**) survival as a percentage. Kruskal–Wallis test was performed, followed by the Dunn’s post hoc test, comparing different concentrations versus 0 mg/mL HAE-*Pq* per genotype. *p* < 0.05. Color code in schematic diagrams: α-syn overexpression period (red rectangle), HAE-*Pq* exposure period (olive green rectangle), larval and adult stages are indicated (light blue).

The next in vivo assay consisted of determining the effect of HAE-*Pq* on the viability of control and PD flies at both larval and adult stages. Larval viability was determined by administering 0, 0.1, or 1 mg/mL HAE-*Pq* during the larval period and quantifying the number of adult flies that emerged (Figure 8a). The number of emerging adult control flies developed in 0.1 mg/mL HAE-*Pq* was significantly higher than age-matched unexposed flies (Figure 8b; *p* = 0.04). This increase was not observed at 1 mg/mL of extract (*p* = 0.74). Similar results were observed in PD flies, i.e., the number of emerged adult flies was significantly higher at 0.1 mg/mL (*p* = 0.003), but this effect was not observed at 1 mg/mL (Figure 8c; *p* = 0.10). The viability assay conducted in adult flies consisted of administering HAE-*Pq* during 7 days of the adult stage, and the number of death events was recorded every 24 h (Figure 8d). No toxic effects of HAE-*Pq* were detected in control flies, where no death events occurred during the assay (Figure 8e). In PD flies, only one death event was recorded, and statistical analysis indicated no significant differences at 0.1 (*p* = 0.44) or 1 mg/mL HAE-*Pq* (Figure 8f; *p* > 0.99). Altogether, these results indicate that HAE-*Pq* is not aversive to flies and exhibits no toxicity during larvae and adult stages at 0.1 and 1 mg/mL of extract, supporting its use in the next experiments.

### 3.6. Effects of HAE-Pq on Motor Performance of Female Control and PD Flies

Transgenic α-syn fly models reproduce the progressive motor impairment associated with dopaminergic neuron degeneration [61,62]. Previous studies have described this motor decline in male PD flies overexpressing the codon-optimized human α-syn sequence in dopaminergic neurons, unveiling an age-dependent reduction in motor response.

**Figure 9:**
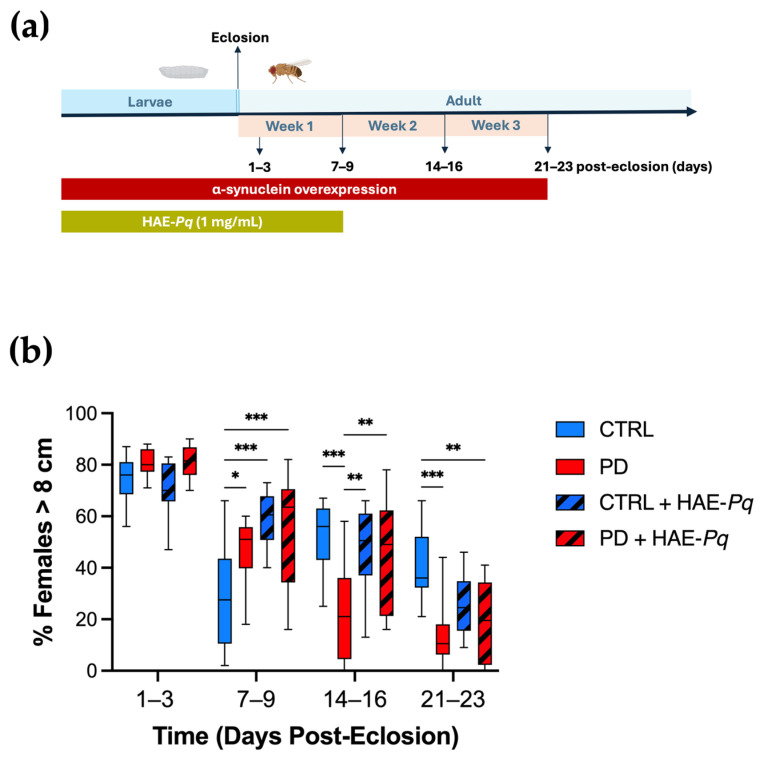
Effects of HAE-*Pq* on motor performance in control and PD flies. (**a**) Schematic representation of the experimental design for the climbing assay. The timeline indicates the stages of the fly’s life cycle: larval phase (blue) and adult phase (light blue) shown in days post-eclosion. Overexpression of α-syn encompasses both larval development and the adult stage (red bar). HAE-*Pq* (1 mg/mL) was administered throughout the larval stage and continued until one–week post–eclosion (olive green bar). Locomotion assays were performed on adult flies at 1–3, 7–9, 14–16, and 21–23 days post–eclosion. (**b**) Data are presented as the interquartile range with maximum and minimum ranges of the percentage of females that escaped above the 8 cm threshold during 10 s. Statistical analysis was performed using two–way ANOVA followed by Tukey’s post hoc test (*n* = 10), comparing the percentage of females surpassing the 8 cm climbing threshold across different genotypes and treatments. * *p* < 0.05, ** *p* < 0.01, and *** *p* < 0.001. Color code: CTRL, control flies unexposed (blue solid box); PD, PD flies unexposed (red solid box); CTRL + HAE-*Pq*, control flies exposed to 1 mg/mL of HAE (blue hatched box); PD + HAE-*Pq*, PD flies exposed to 1 mg/mL of HAE (red hatched box).

We characterized the motor performance of female control and PD flies at 1–3, 7–9, 14–16, and 21–23 days post-eclosion using the climbing assay (Figure 9a). No significant differences in climbing ability were observed between control and PD females at 1–3 days post-eclosion (*p* = 0.80). A transient significant major motor response was detected in PD flies at 7–9 days post-eclosion (*p* = 0.05). However, from 14 to 16 days post-eclosion onwards, PD flies exhibited a significantly reduced motor response in comparison with control individuals (Figure 9b, blue vs. red boxes, *p* < 0.001). These results indicate that female flies overexpressing human α-syn in dopaminergic neurons recapitulate the progressive decline in motor performance, exhibiting a pre-motor phase followed by a motor impairment phase from 14 to 16 days onward.

Since 1 mg/mL of HAE-*Pq* did not alter the food intake and was the highest concentration evaluated in viability assays, it was selected for administration through the standard fly diet. Given that α-syn overexpression in this PD model begins from early stages of the fly life cycle, the scheme of HAE-*Pq* administration covered from the embryonic stage until 7–9 days post–eclosion (Figure 9a). Control flies exposed to HAE-*Pq* exhibited a significantly higher motor response at 7–9 days compared with age-matched unexposed controls (Figure 9b, *p* < 0.001). Interestingly, withdrawal of the extract resulted in flies with motor performances similar to those of control flies never exposed to the extract (14–16 days: *p* = 0.88 and 21–23 days: *p* = 0.09), indicating that this effect was reversible and directly dependent on the continuous presence of HAE-*Pq*. In PD flies, the administration scheme of HAE-*Pq* prevented the decline in motor response by 14–16 days. These flies presented a climbing performance similar to unexposed control flies (*p* = 0.76) and significantly higher than unexposed PD flies (Figure 9b, *p* = 0.003). Notably, this effect did not persist at 21–23 days old, as exposed PD flies exhibited similar motor performance to unexposed PD animals (*p* = 0.91).

Altogether, these results indicate that 1 mg/mL of HAE-*Pq* induces a transient hyperlocomotion in young control flies, while the same scheme of administration delays the onset of motor impairment in female PD flies by one week, persisting for up to one week after discontinuing the extract exposure. This provides preliminary evidence of a possible neuroprotective effect of HAE-*Pq* in this genetic PD animal model.

### 3.7. Effects of HAE-Pq on Dopaminergic Neurons of Female Control and PD Flies

**Figure 10:**
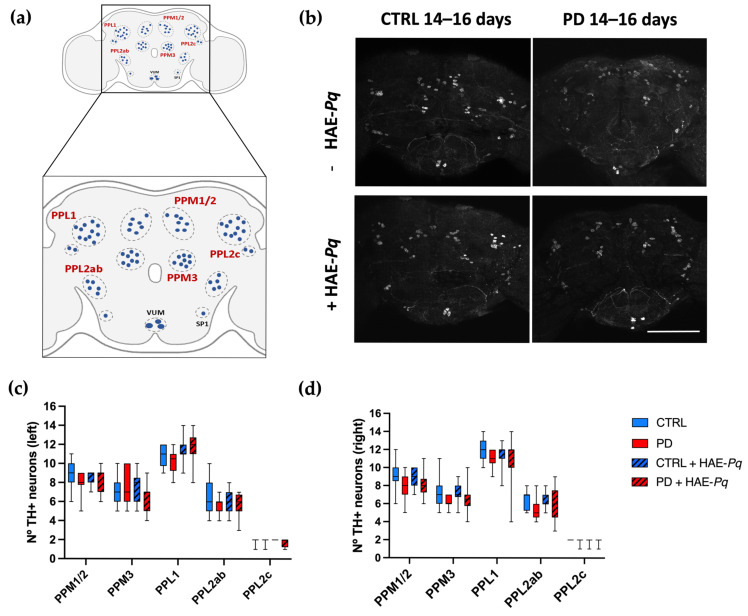
Effects of HAE-*Pq* on dopaminergic neurons in control and PD flies aged 14–16 days. (**a**) A representative scheme illustrating the dopaminergic cluster distribution in the adult posterior part of the fly brain: protocerebral posterior medial 1/2 (PPM1/2), protocerebral posterior medial 3 (PPM3), protocerebral posterior lateral 1 (PPL1), protocerebral posterior lateral 2c (PPL2c), and protocerebral posterior lateral 2ab (PPL2ab). (**b**) Representation of the TH+ neurons identified in the posterior region of the female fly brain in control and PD flies, unexposed and exposed to 1 mg/mL HAE-*Pq*. Scale bar: 100 μm. Quantifications of TH+ neurons in the left (**c**) and right (**d**) brain hemispheres of each experimental group. Data are presented as the interquartile range with maximum and minimum ranges of quantifications in 10–15 brains per experimental group. A two–way ANOVA was conducted, followed by Tukey’s post hoc test, to compare the experimental groups for each cluster. *p* < 0.05. Color code: CTRL, control flies unexposed (blue solid box); PD, PD flies unexposed (red solid box); CTRL + HAE-*Pq*, control flies exposed to 1 mg/mL of HAE (blue hatched box); PD + HAE-*Pq*, PD flies exposed to 1 mg/mL of HAE (red hatched box).

Similarly to mammals, in *Drosophila*, motor performance relies on the integrity of the dopaminergic system [75,76,77]. In flies, somas of dopaminergic neurons are distributed symmetrically in different clusters in the anterior and posterior parts of the brain (Figure 10a). The integrity of dopaminergic neurons in *Drosophila* may be analyzed by identifying and quantifying the neuronal soma. To assess whether dopaminergic neuron loss underlies the motor impairment observed in PD female flies and to determine the effect of HAE-*Pq*, we quantify the number of TH+ neurons in control and PD flies, either unexposed or exposed to 1 mg/mL HAE-*Pq*.

Quantifications were performed at 7–9 days (pre-motor phase) and 14–16 days of age (motor impairment period). At 7–9 days old, no differences were observed in the number of PPM1/2 (left, *p* > 0.99; right, *p* = 0.54), PPM3 (left, *p* > 0.99; right, *p* > 0.99), PPL1 (left, *p* > 0.99; right, *p* > 0.99), PPL2ab (left, *p* > 0.99; right, *p* > 0.99), and PPL2c (left, *p* > 0.99; right, *p* > 0.99) between control and PD female flies in either brain hemisphere (Figure S4c,d). The administration of HAE-*Pq* did not change the number of dopaminergic neurons in any cluster of both control and PD flies at 7–9 days old. This result indicates that the hyperlocomotion observed in control flies exposed to HAE-*Pq* during development is not attributable to an increased number of dopaminergic neurons.

The same analysis carried out at 14–16 days old demonstrated that both control and PD flies do not exhibit differences in the number of dopaminergic neurons in either the left (PPM1/2, *p* = 0.74; PPM3, *p* > 0.99; PPL1, *p* > 0.99; PPL2ab, *p* = 0.65; and PPL2c, *p* > 0.99) or right hemisphere (PPM1/2, *p* = 0.37; PPM3, *p* = 0.36; PPL1, *p* = 0.91; PPL2ab, *p* = 0.63; and PPL2c, *p* > 0.99) (Figure 10b–d). Thus, the motor impairment displayed by PD females at 14–16 days is not associated with dopaminergic soma loss. Control and PD flies exposed to 1 mg/mL HAE-*Pq* did not exhibit differences in the number of dopaminergic neurons compared to age-matched unexposed individuals, indicating the extract does not alter the dopaminergic soma integrity in both genotypes under the tested conditions.

## 4. Discussion

This study represents the first step in evaluating the effect of *P. quadrangularis* in a neuronal cell line and in a neurodegenerative context, using a genetic *Drosophila* PD model that recapitulates progressive motor impairments and dopaminergic neurodegeneration. This study supports and reinforces the previously reported antioxidant capacities of *Pq* and provides preliminary evidence consistent with a potential in vivo neuroprotective effect.

In this work, we obtained HAE and four subfractions from *Pq* and characterized their phytochemical composition and in vitro antioxidant capacity using a standardized battery of antioxidant assays. Overall, HAE-*Pq* showed consistent in vitro antioxidant activity compared to its subfractions, supporting its selection for subsequent exploratory assays in cell culture and in vivo experiments. Previously, the phytochemical composition of *Pq* revealed the presence of 37 compounds in HAE, including tremetones, coumarins, flavonoids, and phenolic acids [51]. In this study, UHPLC-MS analysis showed the presence of 53 metabolites in HAE-*Pq,* with 16 additional metabolites reported. The differences observed in the composition of *Parastrephia* extracts, determined by UHPLC-MS, could be closely related to ecological and geographic factors specific to the collection areas. Altitude, solar radiation, water availability, soil quality, and temperature are environmental variables that directly influence the synthesis and accumulation of secondary metabolites in Andean plants. Furthermore, harvesting at different times of the year involves phenological variations (flowering, fruiting, senescence) that also modify the chemical profiles. Together, these local and seasonal conditions generate a unique metabolic signature in each population, which explains the differences observed between extracts obtained from different collection sites and times.

In this work, the UHPLC-MS analysis unveiled that flavonoids such as jaceidin (36), skullcap flavone II (43) and 5,7,3′,4′-tetrahydroxy-6,8-dimethoxyflavone (32) were present in higher proportions, followed by coumarins esculetin (11) and coumarin glycoside esculin (9). The high flavonoid content of the extract (28 of 53 identified compounds) is consistent with the high TPC and radical scavenging activity demonstrated by ABTS and DPPH assays, together with the high ferric reducing power evidenced by FRAP assay, compared to their subfractions. Importantly, MeOH, EtOAc and DCM subfractions also showed comparable antioxidant capacities, which could also be related to the presence of flavonoids and other phenolic compounds. Future bioassay-guided fractionation studies will be essential to clarify their biological potential and identify the responsible bioactive metabolites.

Cell culture experiments validated the in vitro antioxidant activities of HAE-*Pq* in a biological context. The extract was non-cytotoxic at the tested concentrations for SH-SY5Y cells and triggered a significant reduction in basal ROS levels, from 25 to 200 µg/mL. Interestingly, this response significantly attenuates the increase in ROS induced by the challenge of increasing concentrations of H_2_O_2_. The strong radical scavenging activity of HAE-*Pq*, evidenced by the in vitro antioxidant assays and possibly attributable to its high flavonoid content, is consistent with its ability to attenuate H_2_O_2_-induced ROS in cells. Additional antioxidant responses should be examined to further support this mechanism of action, although other contributing mechanisms cannot be excluded. Certain flavonoids have been proposed to undergo oxidation in the cellular context, generating mild oxidative signals and triggering adaptive antioxidant responses [78,79,80]. This phenomenon, known as hormesis, refers to the process by which low stressor signals prime the cells to withstand a subsequent major oxidative challenge. Whether flavonoids identified in the HAE-*Pq* could trigger this process remains to be explored.

This is the first study assessing *Pq* using a *Drosophila* model of PD, with the in vivo experiments revealing interesting physiological effects. Like other plants, Andean species have evolved to synthesize volatile chemical cues that attract pollinators and repel predators, ensuring reproduction and survival. Flies possess well-developed olfactory and gustatory systems, which enable them to discriminate between odors and flavors, exhibiting innate preferences or aversions to molecules responsible for sensory perceptions. The modified CAFE assay showed that HAE-*Pq* at the concentration used (0.1 and 1 mg/mL) is non-aversive for control and PD flies, supporting the suitability at these concentrations for testing its biological activity in subsequent behavioral assays. Interestingly, PD flies exhibited a slight tendency to intake higher food volumes than control flies, which was significantly higher at 0.1 mg/mL of extract. This increase in food intake could be related to the disruption of the dopaminergic system in this model or the presence of glycoside metabolites in *Pq*, as the UHPLC–MS analysis demonstrates, thus making the HAE-*Pq* appetitive for flies. In flies, DA regulates appetite and motivation for feeding [81,82] and a preference of sugars that is also found conjugated on the HAE-*Pq*. Previous dietary studies in PD patients show a high sugar intake and a preference for choosing sugar-enriched food [83,84], suggested as an increase in DA levels via insulin secretion to compensate for DA deficits. Whether a similar pathway underlies this effect in PD flies needs to be further explored but underscores the importance of the model in recapitulating some aspect of the disease.

Viability analysis in larval and adult stages showed that HAE-*Pq* is safe for flies at the tested concentrations, as no changes in the number of emerged adult flies and no evidence of adult mortality were observed in control and PD flies. An intriguing finding was the significant increase in the number of emergent adult control and PD flies at 0.1 mg/mL HAE-*Pq*, which is not observed at 1 mg/mL. This suggests that larval exposure to low doses of HAE-*Pq*, which is enriched in flavonoids, may provide a genotype-independent developmental advantage. Flavonoids have been reported to influence the reproduction of insects by modulating the feeding behavior, oviposition site preference, and reproduction physiology [85]. These effects depend on the flavonoid type and insect species. Such plant-insect interactions have not been extensively studied in *Drosophila*. Our experimental design for assessing the effect on larval viability included a mating scheme where parents were exposed to HAE-*Pq* for 24 h, which resulted in both parents (acute exposition) and offspring (chronic exposition) being exposed to the extract. Under these conditions, the effect observed at low doses of flavonoids may provide antioxidant protection for reproductive tissues and gametes that results in increasing the number of embryos oviposited and, as a consequence, leading to an increase in adult emergence. Whether hormesis underlies this effect remains an open question for future studies. Interestingly, the therapeutic applications of flavonoids in male reproductive disorders have been proposed in humans. In this context, studies have shown that quercetin, rutin, and other flavonoids may benefit spermatogenesis and sperm quality, which could be related to their antioxidant activities as well as to the modulation of other cellular pathways [86]. However, future studies should address this effect in *Drosophila*, the mechanisms involved, and whether plants from extreme environments could serve as therapeutic agents for reproductive disorders.

In this study, we employed a genetic fly PD model with overexpression of human α-syn codon-optimized sequence in dopaminergic neurons. This codon-optimized sequence has been expressed in photoreceptors, exhibiting early synaptic degeneration followed by functional abnormalities in electroretinograms (ERG) at late stages, which progresses with age [59]. This sequence has also been expressed in dopaminergic neurons, recapitulating the impaired motor response correlated with the loss of dopaminergic neurons in PPL1, PPM1/2, and PPM3 clusters [61,62]. We focused our analysis on female flies, since motor decline and neurodegeneration has been primarily characterized in males. The evaluation of HAE-*Pq* was also performed in females as a first approach to explore its potential neuroprotective effects. Nevertheless, extending these analyses to males will be necessary to assess possible sex-dependent differences in the response to exogenous antioxidants. This is a relevant aspect, as epidemiological data indicate a lower incidence of PD in women than in men, although disease progression and mortality rates may differ between sexes [87]. Evidence from rodent studies further supports sex differences in the expression of dopaminergic components, maturation, and vulnerability to neurodegeneration, with females often less affected [88,89]. Moreover, recent works in *Drosophila* demonstrated that *park*^−/−^ male flies displayed higher redox imbalance in mitochondria of vulnerable dopaminergic neurons than females, suggesting that brain redox physiology could be influenced by sex and that the effectiveness of antioxidant therapeutic strategies may vary between sexes [90].

Our climbing test results indicated that female PD flies exhibit a pre-motor phase followed by a motor impairment phase. This progressive motor decline has been reported previously in male counterparts [61,62]. During the pre-motor phase, at 7–9 days, females displayed a transient hyperlocomotion, which may be attributable to compensatory adaptation mechanisms in the dopaminergic system prior to motor decline. Interestingly, in previous studies using the same test, this hyperlocomotion phenomenon has not been evidenced in male flies of the same genotype, suggesting possible sex-specific differences in compensatory capacity or dopaminergic circuit regulation [61,62]. Our analysis of dopaminergic neurons unveiled that, in females, motor impairments from 14 to 16 days onwards are not associated with a loss of dopaminergic soma, suggesting this effect could be linked to synaptic terminal dysfunction rather than neuronal loss. This is consistent with the “dying back” hypothesis of neurodegeneration and is supported by previous works overexpressing this sequence in photoreceptors of flies, which demonstrated early synaptic dysfunction [59]. Other fly neurodegenerative models have also observed a display of motor deficit and the absence of dopaminergic soma loss, but changes in the TH signal intensity [91]. In our work, the number of dopaminergic neuron somas was assessed at the pre-motor phase and at the onset of motor impairment, which could represent an early stage of disease progression where functional changes in synaptic terminals are preceding structural alterations. The loss of dopaminergic connectivity should be assessed in this PD animal model, and other preclinical animal models, to better comprehend the successive steps underlying the neurodegenerative process, which begins earlier than when motor impairments are evident. This approach could be valuable for determining whether there are thresholds for neuronal recovery.

The delay in the onset of motor deficit in female PD flies fed a standard diet supplemented with HAE-*Pq* is aligned with the antioxidant activity demonstrated in vitro and in cell culture. Complementary in vivo antioxidant assays will be required to further support this mechanism of action, including the assessment of antioxidant enzymes, non-enzymatic mechanisms, and the ARE/Nrf2 pathway. Moreover, it remains to be assessed whether other pathogenic mechanisms beyond oxidative stress, including mitochondrial integrity, apoptosis, autophagy among others, which contribute to disease progression, could also be modulated by metabolites present in HAE-*Pq* and thereby underlie its neuroprotective effect. In this context, flavonoids such as quercetin, apigenin, and luteolin—also detected in the HAE-*Pq*—are known to modulate intracellular pathways, including PI3K/Akt/GSK-3β, Nrf2/ARE, JNK/p38, NF-kB cascades, and BDNF/Trk signaling [21,22,23,24,25,26,27,28,29,30,31,32,33,34,35,36,37,38,39,40]. Thus, other related flavonoids in major abundance, such as jaceidin, skullcapflavone II, or 5,7,3′,4′-tetrahydroxy-6,8-dimethoxyflavone, may likewise modulate inflammatory responses, neurotrophic signaling, and cell survival pathways. Interestingly, a previous study on skullcapflavone II has demonstrated antioxidant and anti-inflammatory activities in cell cultures and its ability to inhibit the aggregation of α-syn, preserve the neurite length, and DA content in SH-SY5Y cells [92,93]. Regarding jaceidin and 5,7,3′,4′-tetrahydroxy-6,8-dimethoxyflavone, their studies in neuroprotection are limited. However, jaceosidin, a related flavone, has shown neuroprotection in an animal model of allergic encephalomyelitis by suppressing microglia inflammatory activation [94]. Additionally, the coumarin esculetin, which was the second metabolite in major abundance in the HAE-*Pq*, has shown bifunctional antioxidant activity and neuroprotection in the SH-SY5Y cell culture model of Alzheimer’s disease (AD) [95] and in Huntington’s disease cell culture and transgenic *Drosophila* models [96]. Thus, the positive effects of HAE-*Pq* in the α-syn overexpressing flies might be the result of multimodal mechanisms whereby polyphenolic compounds (flavonoids and coumarins) act in concert or synergistically to yield neuroprotection. However, direct evidence for such synergy remains to be established. In this context, future studies should therefore expand toward assessing inflammatory responses in PD models, since neuroinflammation is a well-recognized contributor to PD pathogenesis and α-syn dysfunction or overexpression has been associated with increased neuroinflammatory markers [97,98,99]. Here, *Drosophila* may be a suitable model for this exploratory purpose, allowing the evaluation of conserved immune-related pathways such as relish (the NF-kB homolog in flies), nitric oxide, and nitric oxide synthase activity, which has previously been studied in the context of flavonoid bioactivity [100].

The concentrations of HAE tested in flies in our study fall within the range commonly assessed in related studies [101,102,103,104,105]; however, future studies should expand the range of concentrations evaluated. The administration scheme employed in this work suggests that HAE-*Pq* may exert a preventive rather than a restorative effect, as the extract was supplemented simultaneously with α-syn overexpression. Once the extract was withdrawn, PD flies exhibited motor deficits one week later, which is consistent with the sustained α-syn overexpression that progresses as flies age. This finding raises the possibility that prolonging the administration scheme of HAE-*Pq* could further delay the onset of motor phenotypes, an aspect that should be examined in depth, as well as other doses and treatment windows. Moreover, future analyses should also include the administration of a comparative plant extract with reported neuroprotective effects, since the complex mixture of metabolites present in such extracts provides a more relevant and useful benchmark for evaluating the efficacy of *Pq*. Isolated flavonoids or defined mixtures could also serve as valuable comparative references, but their selection should be carefully made according to the experimental context.

Finally, results in control flies demonstrated that HAE-*Pq* is non-neurotoxic for the dopaminergic system, at least at the level of soma integrity. The exposition scheme implemented in our work covered the neurodevelopmental period and the first week post-eclosion, which has been termed as a “critical period” in *Drosophila*, where maturation and refinement of certain neuronal circuits in the central nervous system take place [106,107,108,109]. The hyperlocomotion observed at 7–9 days may be the result of functional effects on the dopaminergic system, but this possibility needs to be further explored. Notably, this effect did not persist after the extract withdrawal, reflecting a direct effect of HAE-*Pq*. It is important to note that our study focused primarily on behavioral outcomes. Further investigations, including additional behavioral paradigms, signaling pathway analysis, and neuronal integrity assessment—particularly at the synaptic terminal level—will be required to better establish the neuroprotective effect of *Pq* and to confirm that HAE-*Pq* does not display negative impacts in the short and long term. Moreover, *Drosophila* offers a valuable in vivo screening platform for identifying potential neuroprotective agents, but it has inherent translational constraints compared to mammalian systems. Validation in mammalian models will therefore be necessary to progress and delineate dose–response relationships, confirm the mechanistic pathways, and support the therapeutic relevance of this extract.

Neurons are particularly susceptible to developing oxidative stress environments. The high ATP demand to support neurotransmission and ionic gradients [110] requires highly active mitochondria, resulting in major ROS production as a metabolic byproduct. Other factors, such as the role of reactive species as signaling molecules at both developmental and mature stages of neuronal life [111,112], the auto-oxidation of neurotransmitters such as dopamine [113,114], the higher content of redox-active transition metals [115], the activated microglia [116] and the limited neuronal antioxidant response [117,118] further contribute to developing pro-oxidant intracellular environments. Thus, genetic or environmental insults may induce neuronal damage and neurodegeneration. In this context, strategies focused on managing reactive species may offer an alternative to prevent neuronal damage, promote healthy aging, and, in turn, prevent the onset of NDs. In the context of NDs, this approach is more therapeutically relevant if the neuroprotective strategy may involve the simultaneous modulation of different pathological mechanisms participating in the progression of the disease. In this context, phytochemicals such as flavonoids may offer a therapeutic multitarget approach.

## 5. Conclusions

This work provides novel in vitro and in vivo evidence of the biological activity of *P. quadrangularis*, a plant traditionally used by high Andean communities. Among the tested extract and subfractions, HAE-*Pq* exhibited the highest phenolic content and consistent in vitro antioxidant activity. Phytochemical profiling unveils that HAE-*Pq* is particularly rich in flavonoids, followed by coumarins and phenolic acids. Notably, jaceidin—the most abundant metabolite in HAE-*Pq*—remains largely unexplored in a neurodegenerative context. The in vitro antioxidant capacity was further validated in a neuronal cell line, SH-SY5Y cells, where HAE-*Pq* reduced basal ROS levels and attenuated H_2_O_2_-induced ROS. Additionally, this study provides the first exploratory evidence of the potential neuroprotective effect of *Pq* in a neurodegenerative animal model using a PD genetic fly model, where the administration of HAE-*Pq* delayed the onset of motor deficits by one week in females. These results underscore the value of *Drosophila* as an initial in vivo platform to screen plant extracts with neuroprotective potential, which should subsequently progress to superior animal models for its further validation. Moreover, together, these findings highlight *P. quadrangularis* as a promising source of bioactive compounds with therapeutic relevance for pathologic conditions in which oxidative process is involved, such as NDs. Importantly, this work provides novel insights that scientifically validate the ethnopharmacological use of plants from the Atacama Desert while underscoring responsible exploration and conservation.

## Figures and Tables

**Table 1:** In vitro antioxidant activities of HAE and subfractions of *P. quadrangularis*.

		Subfractions				Standards	
Antioxidant Assay	HAE	MeOH	EtOAc	DCM	n-hex	Trolox	EDTA
TPC ^a^	804.4 ± 8.6	636.1 ± 6.1	717.4 ± 8.0	555.0 ± 5.4	101.2 ± 4.5	-	-
TFC ^b^	91.9 ± 1.5	100.2 ± 2.4	104.3 ± 2.1	104.8 ± 3.0	101.2 ± 4.5	-	-
FRAP ^c^	201.6 ± 6.1	164.0 ± 0.9	108.9 ± 1.1	58.6 ± 2.8	5.1 ± 1.0	-	-
Chelating ^d^	882.7 ± 1.7	883.1 ± 0.7	N/A	N/A	N/A	-	4.1 ± 1.0
DPPH ^d^	93.5 ± 14.3	106.9 ± 2.0	109.7 ± 2.5	648.7 ± 158.3	588.9 ± 294.2	7.3 ± 0.9	-
ABTS ^d^	9.0 ± 0.1	7.7 ± 0.8	3.8 ± 0.4	10.4 ± 0.2	>100	9.6 ± 0.5	-

^a^ Expressed in mg gallic acid equivalent/g dry extract; ^b^ Expressed in mg quercetin equivalent/g dry extract; ^c^ Expressed in mg Trolox equivalent/g dry extract; ^d^ IC_50_ expressed in μg/mL; N/A: No Activity.

**Table 2:** UHPLC–ESI–QTOF–MS Identification of metabolites presents in the HAE-*Pq*.

Compound No. ^a^	Retention Time (min.)	Measured Mass ^b^ (*m*/*z*)^+/−^	Molecular Mass (*m*/*z*)	Molecular Formula	Tentative Identification	Metabolite Type	Signal Intensity
1	0.61	325.0927	324.0854	C_15_H1_6_O_8_	Skimmin	Coumarin glycoside	10,972
2	0.63	353.0878	354.0951	C_16_H_18_O_9_	Chlorogenic acid	Phenolic acid	39,926
3	0.65	147.0300	148.0374	C_5_H_8_O_5_	Citramalate	Carboxylic acid	7329
4	0.77	175.1197	174.1124	C_6_H_14_N_4_O_2_	L-Arginine	Amino acid	867
5	0.79	121.0292	122.0365	C_7_H_6_O_2_	4-Hydroxybenzaldehyde	Hydroxybenzaldehyde	16,954
6	0.81	133.0144	134.0217	C_4_H_6_O_5_	Malic acid	Carboxylic acid	61,586
7	1.02	163.0396	164.0468	C_9_H_8_O_3_	4-Coumaric acid	Phenolic acid	65,359
8	1.07	341.0874	340.0801	C_15_H_16_O_9_	Sculin hydrate	Coumarin glycoside	5025
9	1.11	339.0716	340.0789	C_15_H_16_O_9_	Sculin	Coumarin glycoside	126,353
10	1.15	191.0403	192.0475	C_10_H_8_O_4_	Scopoletin	Coumarin	7209
11	1.74	177.0193	178.0266	C_9_H_6_O_4_	Esculetin	Coumarin	186,459
12	1.86	609.142	610.1493	C_27_H_30_O_16_	Rutin	Flavonoid	5116
13	2.00	693.1953	694.2026	C_32_H_38_O_17_	Helonioside A	Phenylpropanoids	3956
14	2.31	593.1515	594.1588	C_27_H_30_O_15_	Nicotiflorin	Flavonoid	7593
15	2.45	193.0500	192.0427	C_10_H_8_O_4_	Isoscopoletin	Coumarin	15,627
16	2.50	515.1184	516.1256	C_25_H_24_O_12_	Cynarin	Phenolic acid	8687
17	2.51	447.0923	448.0996	C_21_H_20_O_11_	Luteolin 4′-O-glucoside	Flavonoid	16,819
18	2.52	477.1047	478.1120	C_22_H_22_O_12_	Isorhamnetin 3-galactoside	Flavonoid	4960
19	2.54	207.0656	206.0583	C_11_H_10_O_4_	Escoparone	Coumarin	3136
20	2.59	187.0974	188.1047	C_9_H_16_O_4_	Azelaic acid	Fatty acids	11,150
21	2.61	529.1340	530.1413	C_26_H_26_O_12_	1-Feruloyl-5-caffeoylquinic acid	Phenolic acid	8535
22	2.70	623.1590	624.1663	C_28_H_32_O_16_	5,7-dihydroxy-2-(4-hydroxy-3-methoxyphenyl)-3-[3,4,5-trihydroxy-6-[[(2R,3R,4R,5R,6S)-3,4,5-trihydroxy-6-methyloxan-2-yl]oxymethyl]oxan-2-yl]oxychromen-4-one	Flavonoid	57,574
23	2.77	515.1177	516.125	C_25_H_24_O_12_	3,4-di-O-caffeoylquinic acid	Phenolic acid	22,348
24	2.87	447.0920	448.0993	C_21_H_20_O_11_	4-(3,4-dihydroxyphenyl)-7-hydroxy-5-[(2S,3R,4S,5S,6R)-3,4,5-trihydroxy-6-(hydroxymethyl)oxan-2-yl]oxychromen-2-one	Flavonoid	8863
25	3.13	193.0500	194.0573	C_10_H_10_O_4_	3-hydroxy-4-methoxycinnamic acid	Phenolic acid	107,274
26	3.24	161.0243	162.0315	C_9_H_6_O_3_	Umbelliferone	Coumarin	6960
27	3.52	301.0347	302.0419	C_15_H_10_O_7_	Quercetin	Flavonoid	15,657
28	3.58	285.0398	286.0470	C_15_H_10_O_6_	Luteolin	Flavonoid	55,510
29	3.59	315.0501	316.0574	C_16_H_12_O_7_	Isorhamnetin	Flavonoid	38,956
30	3.65	177.0550	178.0622	C_10_H_10_O_3_	4-Methoxycinnamic acid	Phenolic acid	38,855
31	3.82	317.0656	316.0584	C_16_H_12_O_7_	Eupafolin	Flavonoid	5403
32	3.85	345.0616	346.0689	C_17_H_14_O_8_	5,7,3′,4′-Tetrahydroxy-6,8-dimethoxyflavone	Flavonoid	133,795
33	4.22	299.0553	300.0626	C_16_H_12_O_6_	Scutellarein 4′-methyl ether	Flavonoid	23,161
34	4.26	269.0447	270.052	C_15_H_10_O_5_	Apigenin	Flavonoid	12,042
35	4.30	329.0649	330.0722	C_17_H_14_O_7_	(2Z)-4,6-dihydroxy-2-[(4-hydroxy-3,5-dimethoxyphenyl)methylidene]-1-benzofuran-3-one	Aurone	13,100
36	4.48	359.0752	360.0825	C_18_H_16_O_8_	Jaceidin	Flavonoid	423,641
37	4.67	331.0812	330.0739	C_17_H_14_O_7_	Tricin	Flavonoid	12,042
38	4.76	359.0754	360.0827	C_18_H_16_O_8_	Irigenin	Flavonoid	90,973
39	4.81	389.0858	390.0931	C_19_H_18_O_9_	Scaposin	Flavonoid	27,531
40	4.96	147.0437	146.0364	C_9_H_6_O2	4H-chromen-4-one	Chromene	26,034
41	5.10	299.0552	300.0624	C_16_H_1_2O_6_	Diosmetin	Flavonoid	6328
42	5.14	389.0854	390.0927	C_19_H_18_O_9_	5,7,3′-Trihydroxy-3,6,4′,5′-tetramethoxyflavone	Flavonoid	97,985
43	5.33	375.1067	374.0994	C_19_H_18_O_8_	Skullcapflavone II	Flavonoid	172,996
44	5.62	345.0963	344.0890	C_18_H_16_O_7_	Eupatorin	flavonoid	61,057
45	5.72	147.0435	146.0362	C_9_H_6_O_2_	Coumarin	Coumarin	6840
46	5.78	343.0810	344.0883	C_18_H_16_O_7_	Eupatilin	Flavonoid	61,057
47	5.88	283.0605	284.0678	C_16_H_12_O_5_	Acacetin	Flavonoid	5982
48	5.97	299.0543	300.0616	C_16_H_12_O_6_	Kaempferol-4′-methyl ether	Flavonoid	3263
49	6.15	405.1170	404.1097	C_20_H_20_O_9_	5-Hydroxyauranetin	Flavonoid	35,239
50	6.24	313.0699	314.0772	C_17_H_14_O_6_	Cirsimaritin	Flavonoid	6738
51	6.33	363.1222	364.1295	C_22_H_20_O_5_	p-Coumaroyloxytremetone	Tremetone	76,408
52	6.54	359.1111	358.1038	C_19_H_18_O_7_	Gardenin B	Flavonoid	7531
53	6.88	389.1219	388.1146	C_20_H_20_O_8_	3′-Demethylnobiletin	Flavonoid	15,298

^a^ The chemical structure of each compound is found in Figure 6 and Supplementary Material Figure S2. ^b^ The *m*/*z* values were obtained in negative [M−H]^−^ and positive [M−H]^+^ mode.

## Data Availability

The data presented in this study are available from the corresponding author upon reasonable request.

## Supplementary Materials

The following supporting information can be downloaded at: https://www.mdpi.com/article/10.3390/antiox14101226/s1, Figure S1: Modified CAFE assay experimental setup; Figure S2: Chemical structures of the different compounds found by UHPLC-ESI-QTOF-MS analysis and identified in the HAE-*Pq* extract; Figure S3: Effects of HAE-*Pq* on food intake in control and PD flies aged 4–7 days; Figure S4: Effects of HAE-*Pq* on dopaminergic neurons in control and PD flies aged 7–9 days.
