# Supplementary material for: In Vitro Antioxidant Activity and In Vivo Neuroprotective Effect of Parastrephia quadrangularis in a Drosophila Parkinson’s Disease Model
Source: Antioxidants (Basel). 2025 Oct 12;14(10):1226. doi: 10.3390/antiox14101226 (PMC12561120; doi:10.3390/antiox14101226)
Supplement: Supplementary file 1 [file antioxidants-14-01226-s001.zip › antioxidants-3894148-supplementary.pdf]

This PDF includes:

Supplemental figures S1 to S4

Titles and legends for Supplemental figures S1 to S4

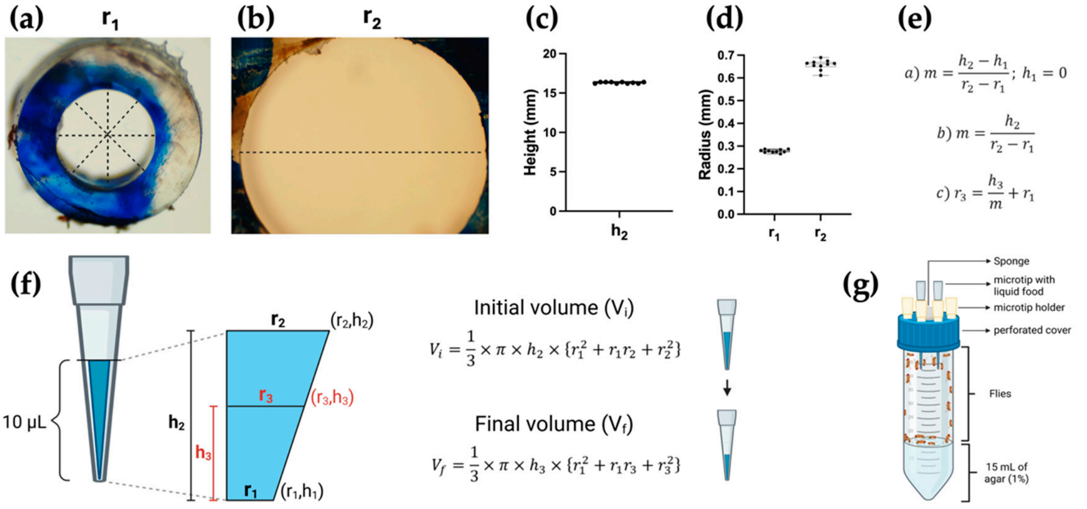

Supplementary figure S1. Modified CAFE assay experimental setup. (a) Representative image showing the inner radius at the base of a microtip. (b) Representative image of the inner radius at the meniscus formed by 10 µL of liquid food (h_2). (c) Mean height (in mm) at the meniscus formed with 10 µL of liquid food in 10 microtips. (d) Mean values of r_1 and r_2. (e) Linear equation used to calculate the slope (m) and r_3. (f) Schematic representation of a microtip containing 10 µL of liquid food indicating the variables h_1, h_2, h_3, r_1, r_2, and r_3, which were incorporated into the truncated cone equation to calculate initial (V_i) and final (V_f) volumes. (g) Schematic representation of the experimental setup in a conic tube containing 15 mL of 1% agar, two microtips with 10 µL of liquid food, and a population of flies.

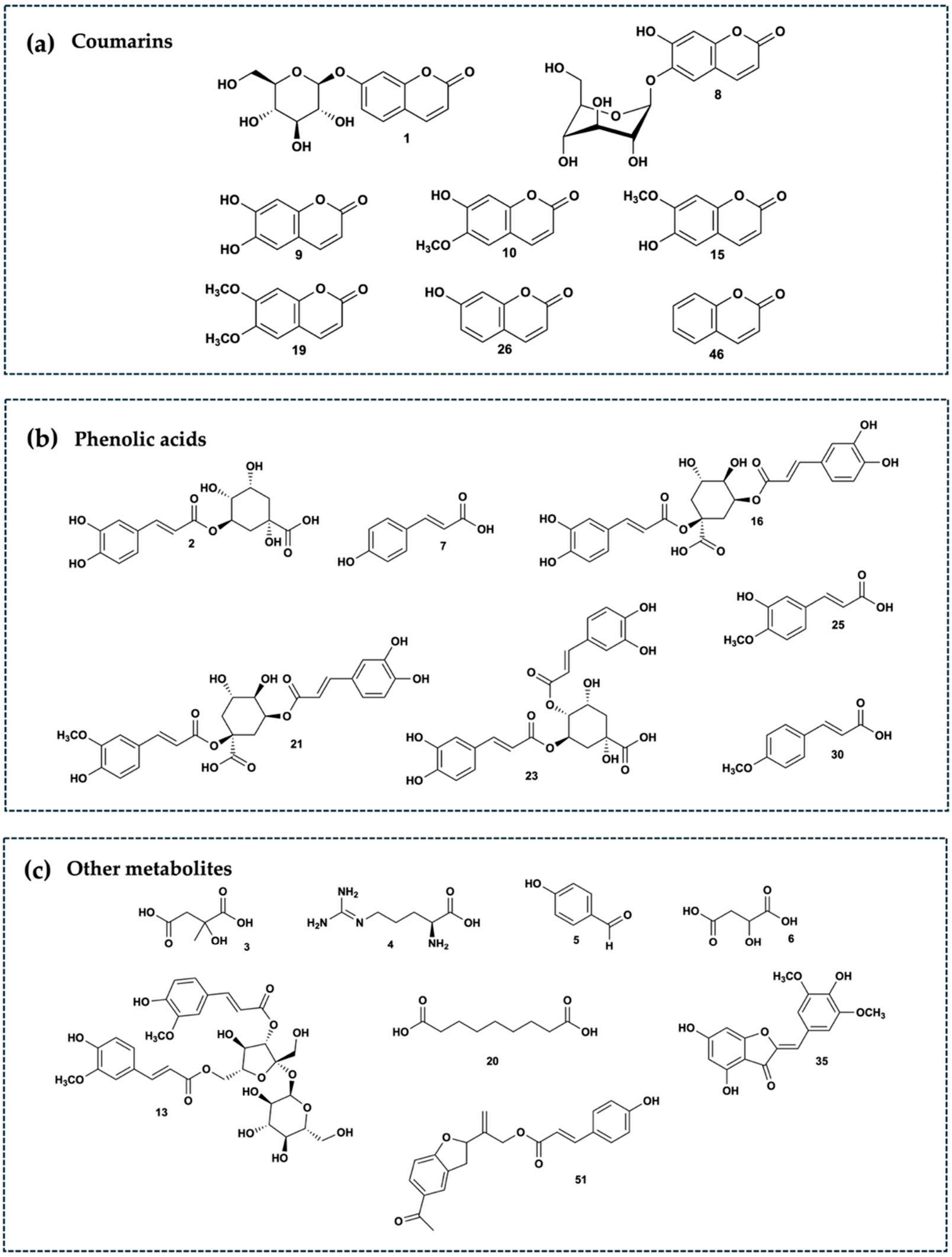

Supplementary figure S2. Chemical structures of the different compounds found by UHPLC–ESI–QTOF–MS analysis and identified in the HAE-*Pq* extract. These are grouped according to their chemical families as coumarins (a), phenolic acids (b), and other metabolites (amino acids, aurones, tremetones, phenylpropanoids, aldehydes, organic acids and fatty acids) (c). The structures of the remaining metabolites identified can be seen in Figure 6.

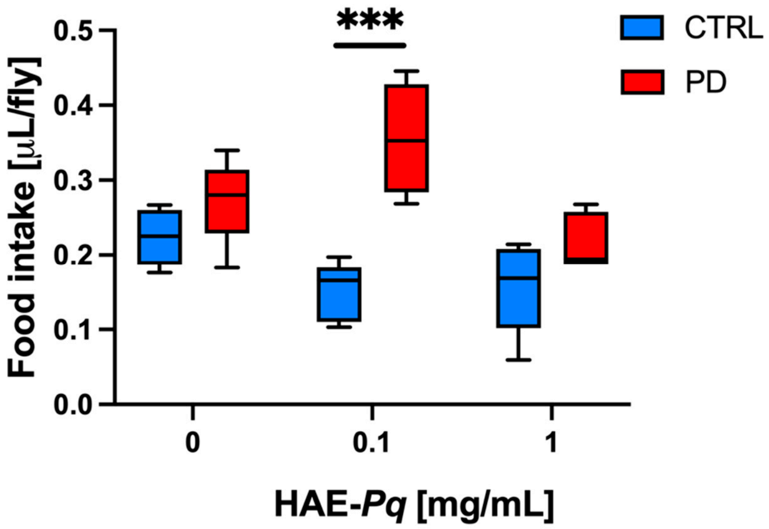

Supplementary figure S3. Effects of HAE-*Pq* on food intake in control and PD flies aged 4–7 days. Adult flies were exposed to a standard diet supplemented with increasing concentrations of HAE-*Pq* (0, 0.1, 1 mg/mL) for 4 h at 25°C. The data are presented as the interquartile range with maximum and minimum ranges of food intake in microliters per fly. A two–way ANOVA was performed, followed by the Bonferroni post hoc test (n=5), comparing different concentrations of HAE-*Pq* versus 0 mg/mL HAE-*Pq* per genotype. ***$p < 0.001$. Color code: CTRL (blue box) and PD (red box).

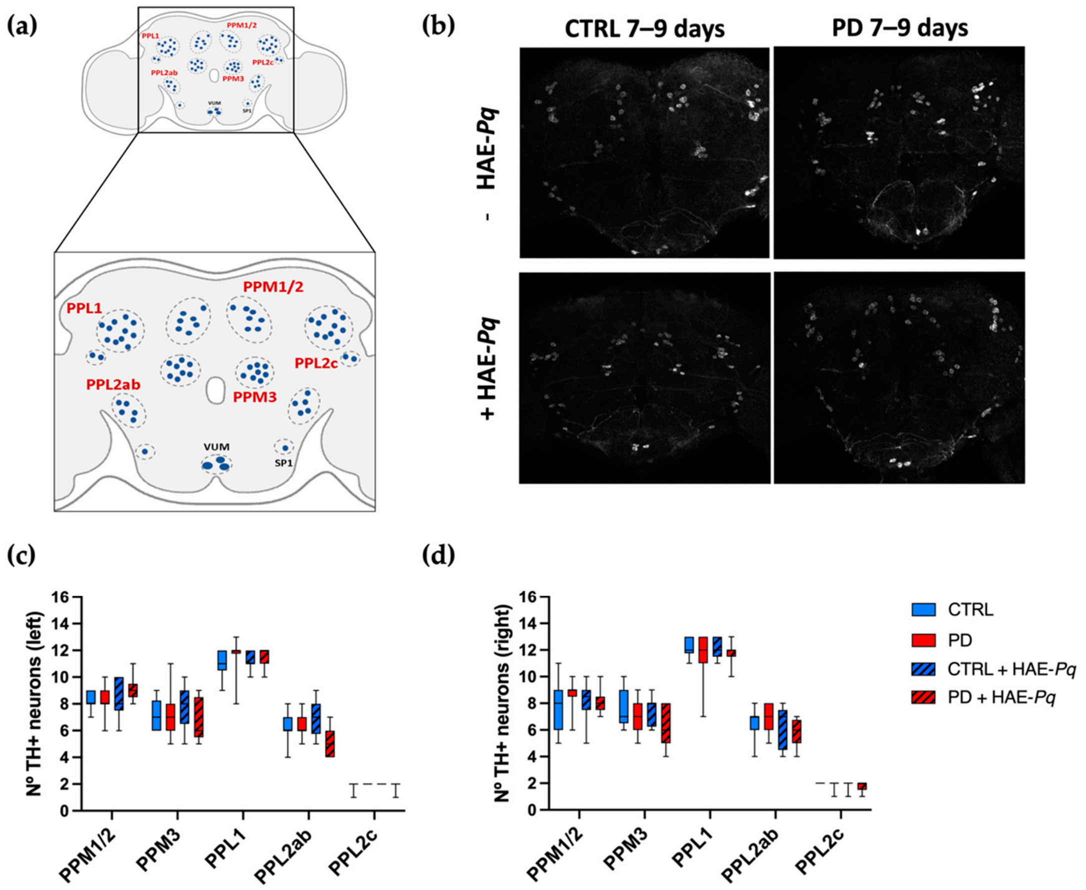

Supplementary figure S4. Effects of HAE-*Pq* on dopaminergic neurons in control and PD flies aged 7–9 days. (a) A representative scheme illustrating the dopaminergic cluster distribution in the adult posterior part of the fly brain: protocerebral posterior medial 1/2 (PPM1/2), protocerebral posterior medial 3 (PPM3), protocerebral posterior lateral 1 (PPL1), protocerebral posterior lateral 2c (PPL2c), and protocerebral posterior lateral 2ab (PPL2ab). (b) Representation of the TH+ neurons identified in the posterior region of the female fly brain in control and PD flies, unexposed and exposed to 1 mg/mL HAE-*Pq*. Quantifications of TH+ neurons in the left (c) and right (d) brain hemispheres of each experimental group. Data are presented as the interquartile range with maximum and minimum ranges of quantifications in 10-15 brains per experimental group. A two–way ANOVA was conducted, followed by Tukey's *post hoc* test, to compare the experimental groups for each cluster. $p < 0.05$. Color code: CTRL, control flies unexposed (blue solid box); PD, PD flies unexposed (red solid box); CTRL + HAE-*Pq*, control flies exposed to 1 mg/mL of HAE (blue hatched box); PD + HAE-*Pq*, PD flies exposed to 1 mg/mL of HAE (red hatched box).
